# Individual residency behaviours and seasonal long-distance movements in acoustically tagged Caribbean reef sharks in the Cayman Islands

**DOI:** 10.1371/journal.pone.0293884

**Published:** 2023-11-27

**Authors:** Johanna Kohler, Mauvis Gore, Rupert Ormond, Bradley Johnson, Timothy Austin

**Affiliations:** 1 Department of the Environment, Cayman Islands Government, George Town, Cayman Islands; 2 Centre for Marine Biodiversity and Biotechnology, Heriot-Watt University, Edinburgh, Scotland, United Kingdom; 3 Marine Conservation International, Edinburgh, Scotland, United Kingdom; Instituto Portugues do Mar e da Atmosfera, PORTUGAL

## Abstract

Understanding how reef-associated sharks use coastal waters through their ontogeny is important for their effective conservation and management. This study used the horizontal movements of acoustically tagged Caribbean reef sharks (*Carcharhinus perezi*) to examine their use of coastal space around the Cayman Islands between 2009 and 2019. A total of 39 (59.1%) tagged sharks (male = 22, female = 17, immature = 18, mature = 21) were detected on the islands wide network of acoustic receivers. The detection data were used to calculate values of Residency Index (RI), Site-Fidelity Index (SFI) and minimum linear displacement (MLD), as well as for network analysis of individual shark movements to test for differences between demographics, seasons, and diel periods. Sharks were detected for up to 1,598 days post-tagging and some individuals showed resident behaviour but the majority of tagged individuals appear to have been one-off or only occasional transient visitors to the area. Generally, individuals showed strong site-fidelity to different areas displaying linear home ranges of < 20 km. The evidence indicates that there was no pattern of diel behaviour. Tagged sharks generally showed increased movements within and between islands during the summer (April–September), which may be related to breeding activity. Some individuals even made occasional excursions across 110 km of open water > 2,000 m deep between Grand Cayman and Little Cayman. One mature female shark showed a displacement of 148.21 km, the greatest distance reported for this species. The data shows that the distances over which some sharks moved, greatly exceeded the extent of any one of the islands’ marine protected areas indicating that this species may be more mobile and dispersive than previously thought. This study provides support for the blanket protection to all sharks throughout Cayman waters, which was incorporated within the National Conservation Act in 2015.

## Introduction

Reef sharks, such as the Caribbean reef shark (*Carcharhinus perezi*), complete their life-cycles within a coral reef ecosystem [1, 2], making them particularly vulnerable to threats, given that the coastal space use of sharks and anthropogenic activities (e.g. fishing pressure) are likely to overlap. For the effective conservation of sharks, it is vital to understand the distribution and behaviour of local populations in relation to human use of coastal space. Until recently, most of the work addressing these issues has focused on a small number of species, notably the nurse (*Ginglymostoma cirratum*), grey reef (*Carcharhinus amblyrhynchos*), blacktip reef (*Carcharhinus melanopterus*), lemon (*Negaprion brevirostris*), and whitetip reef sharks (*Triaenodon obesus*), and been conducted in only a few locations [3–5]. Despite a substantial research effort directed at these species, basic questions concerning the spatial and temporal patterns of reef shark movement ecology remain [5, 6].

Monitoring shark behaviour remains challenging due to the complex habitat and highly mobile nature. However, acoustic telemetry does not require the study animal to be recaptured and has increasingly been used to investigate the behaviour of large-bodied sharks [7–11]. Passive acoustic telemetry collects presence and absence data from uniquely coded acoustic transmitters (tags) that are detected and logged by stationary hydrophones (receivers). The application of various analyses (e.g. network analysis, home range, residency and site-fidelity indices) to acoustic detection data [2, 11–15] has proved valuable in the assessment of individual movement patterns of different species [16, 17].

Evidence from previous acoustic telemetry studies suggests that Caribbean reef sharks are highly resident and exhibit strong site-fidelity to particular areas [2, 18–21]. Other recent data suggest that this species may be more mobile and dispersive than previously thought [2, 20, 22], with individuals undertaking excursions far beyond the typical, relatively small linear home range (< 20 km) and including occasional long-distance movements (>50 km) across open water [11, 23, 24] up to 700 m deep [25]. Furthermore, this species may play an important role in habitat linkages through both diel and ontogenetic habitat shifts, moving from shallower to deeper water between night and day and with increasing individual size [26–28].

The present study was undertaken in the Cayman Islands on the Caribbean reef shark, one of the most abundant species in the coastal waters of the wider Caribbean, including Cayman [22, 24]. Prior to the beginning of shark research in Cayman in 2009 [24], local knowledge of the species consisted only of catch records [29–31] documenting the decline of shark populations of a former shark fishery (up to the 1960s). Legal shark protection is provided by a network of coastal Marine Protected Areas (MPAs) established in 1986 and specific legislation established in 2015 which protects all shark species within national waters [32]. The local economy is highly dependent on tourism and therefore subject to coastal development and other anthropogenic disturbances including recreational / sport fishing and diving [33–38], both of which, as reported elsewhere [39–42], are likely to impact the local shark population. Better knowledge of the movement patterns of Caribbean reef sharks was therefore required to support on-going protection and conservation efforts directed at this ecologically and socio-economically important species [24].

The aims of this study were to: (1) determine the degree of residency and site-fidelity, (2) assess potential inter-island movements, and (3) examine coastal space use, including differences between demographics, seasons, and diel periods, of Caribbean reef sharks in the Cayman Islands. The results provide essential information for effective local shark conservation and enforcement and for understanding Caribbean reef shark behaviour in the wider Caribbean, including insight in potential connectivity across their range.

## Materials and methods

### Study area

**Fig 1:**
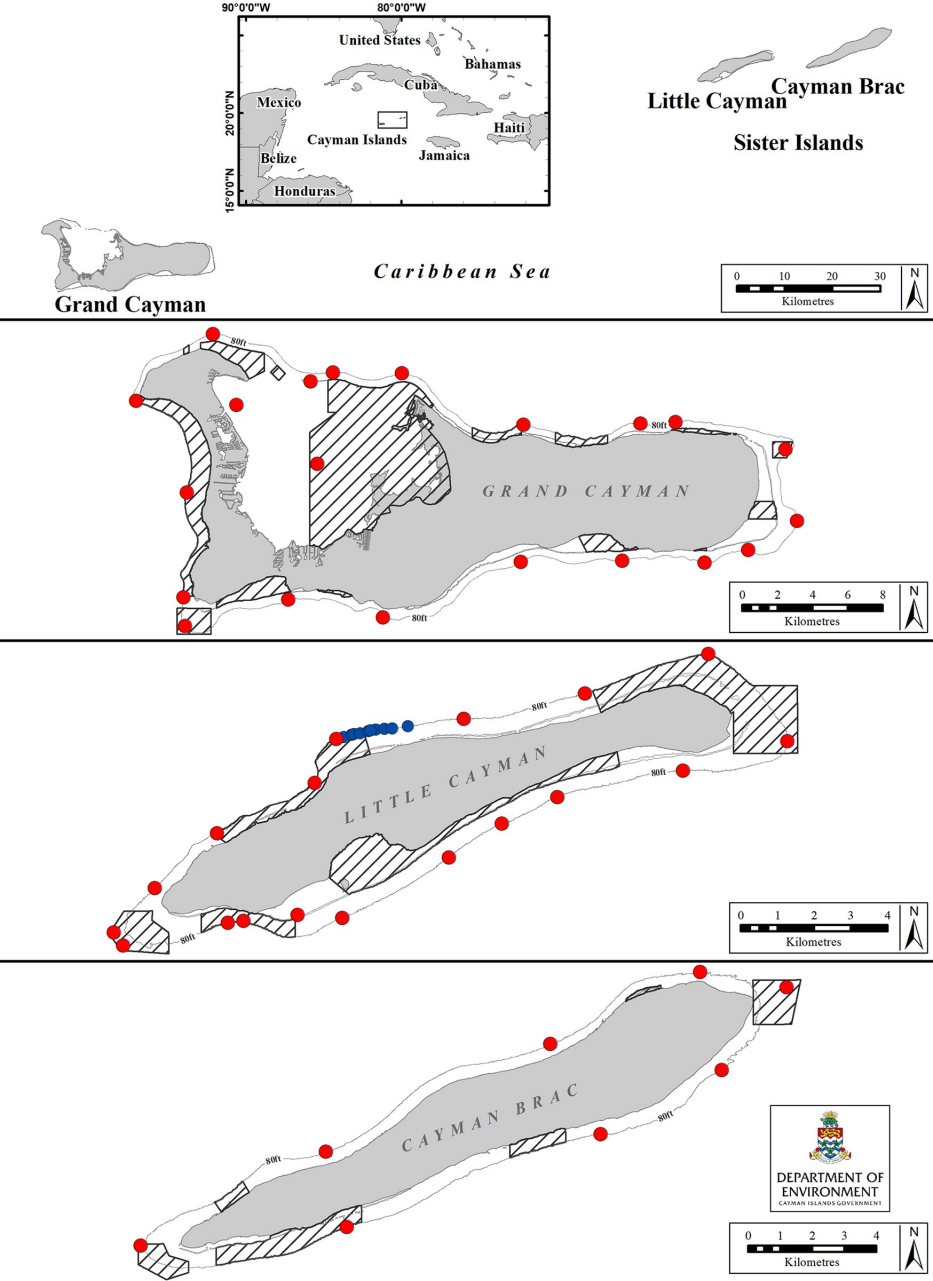
Map showing the location of receiver stations in the Cayman Islands. Red circles indicated receivers (n = 47) belonging to the DoE array and blue circles indicated receivers (n = 10) from the Central Caribbean Marine Institute (CCMI). The line around each island indicates the 25m (85ft) depth contour and shaded areas represent MPAs. Created by the Department of the Environment, Cayman Islands Government. Insert layer’s geography was developed by Esri and sourced from Garmin International, Inc., the U.S. Central Intelligence Agency (The World Factbook), and the National Geographic Society for use as a world basemap [47].

The Cayman Islands, Grand Cayman (19.3222° N, 81.2409° W), Little Cayman (19.6897° N, 80.0367° W) and Cayman Brac (19.7235° N, 79.8017° W), are located in the northwestern Caribbean Sea and cover approximately 264 km^2^ (Fig 1). The distances between Grand Cayman, the largest island, and Little Cayman; and between Little Cayman and Cayman Brac (known locally as the two Sister Islands) are 110 km and 6.5 km, respectively. The marine environment consists of a very narrow coastal shelf, containing various habitats for reef fish [43, 44], edged by a fringing coral reef leading to very steep slopes that reach to more than 2000 m depth [45]. Areas with varying degrees of protection, jointly referred to as Marine Protected Areas (MPAs), covered 43% of the total coastal shelf (45% of Grand Cayman, 48% of Little Cayman and 25% of Cayman Brac; Fig 1) during the time of this study. MPAs have recently been enhanced to a total cover of 55% coastal shelf [46].

### Acoustic array design

Initially, an array of acoustic receivers (VR2W, 69Hz, Innovasea Systems Inc., Canada) was installed by the Cayman Islands Department of the Environment (DoE) along the edge of the coastal shelf in Little Cayman in 2005. Additional receivers were deployed over a period of 11 years, to give a total of 47 receiver stations by 2016 (Grand Cayman = 23, Little Cayman = 18, Cayman Brac = 8; Fig 1). Each receiver was orientated to point downward and attached to a polypropylene rope (at approx. ≥10 m depth) using five heavy-duty cable ties (as per VR2W user manual, Innovasea Systems Inc., Canada). The rope was moored to the substrate (at approx. 25 m depth) and a subsurface float attached to the top of the rope supported the unit and marked the receiver station (S1 Fig). The distance between receivers in the array varied with island size, number of receivers available and bathymetry.

Detection range was tested by suspending a V16 tag in 10–20 m of clear water with slow current and no objects nearby. A VR100 acoustic receiver and handheld GPS was used to determine the maximum distance (250–300 m) at which the emitted signal could still be detected, although in fact the range of the VR100 is not equal to the range of the VR2W’s used in the array. This procedure was repeated 5 times by DoE staff in 2006 and 10 times by MG and RO in 2013. The results suggested, given average local environmental conditions, a detection radius of 300 m, which is within the reported average [2, 48, 49].

It was estimated, assuming all receiver stations were active, that the array covered approximately 7% of the coastal shelf. It should be noted that the signal transmission from the V16 is stronger than that of the smaller V9 tag and that the tag-specific delays between successive signal transmissions could have led to sharks passing a receiver towards the edge of the detection area (diameter 600 m) without being detected. A tagged shark could also pass a receiver without being detected if it was below the edge of the intersection between the shallow coastal shelf and steep descent to deep water, known local as the ‘drop off.’ An average swimming speed of 0.6 shark body lengths per second [50–52] and the conservative speed reported of a grey reef shark in the wild and in tropical water [53] were used to estimate a shark’s average speed in this study. A Caribbean reef shark of 150 cm total length (TL) would need 666 s (11 min) to cross the entire detection area of a receiver (600 m), which is about seven times the nominal delay time in transmission. Therefore, the probability of detecting a shark on receiver stations was considered acceptable. The array was not expected to provide continuous monitoring of shark movements, but rather to detect whether sharks were present within the detection range of a receiver station on any given day.

Independently, the Central Caribbean Marine Institute (CCMI) installed an array of 10 receivers (VR2W, 69Hz, Innovasea Systems Inc., Canada, April 2017–October 2018, Dr Allison Candelmo per. comm.) for lionfish research on Little Cayman (Fig 1). Given that tagged sharks were also detected on that array, these detection data were incorporated into the present analysis.

### Shark capture and tagging

Between 2010 and 2019, Caribbean reef sharks were fitted with tags in the coastal waters of the three Cayman Islands. Capture sites were chosen depending on weather conditions and local knowledge of shark abundance. Sharks were captured either on stationary scientific longlines or on handlines operated directly from the research vessel (S2 Fig). Scientific longlines (500 m) with approximately 50 baited gangions were set from a boat in approximately 15–25 m depth, based on the target species and depending on habitat. The longline was composed of a double braided ¼” (0.64 cm) main line with each end of the line anchored in sand. Gangions were 2.5 m long and composed of a 3/16” heavy duty snap attached to 1.5 m 3/16” (0.48 cm) braided polyester line, attached in turn to 50 cm monofilament (300 lbs / 136 kg test strength), attached to a 40 cm long wire leader ending in a 16/0 circle hook (to reduce the risk of accidental gut-hooking, [54]). Joins in gangions were connected with swivels and monofilament, and the wire leader and circle hook were secured with crimps. Hooks were baited mainly with similar sized pieces of Atlantic Mackerel (*Scomber scombrus*), but alternatively with local tuna (*Thunnus* spp.) or barracuda (*Sphyraena* spp.), depending on availability. Gangions were evenly spaced (≥ 6 m apart) along the mainline together with support buoys. Water depth and GPS location were recorded at the centre point of each longline using a handheld depth sounder (Vexilar Inc., USA) and handheld GPS (Garmin, Kansas) respectively.

Single hook handlines were approximately 15 m long. The line was made of double braided ¼” rope and cleated onto the boat. A baited 16/00 circle hook was attached to the other end and weighted with an approximately 0.5 kg weight. The upper part of the line was held at the surface by a buoy approximately 15 m away from the boat. The boat was either anchored in sand or attached to a mooring. Water depth and location were recorded at the centre of the boat via depth sounder and handheld GPS.

To avoid unnecessary stress to animals captured [55], lines were checked at 20 min intervals to see whether a shark had been captured and to release any by-catch. Any shark captured was attended to immediately. Caribbean reef sharks captured were considered eligible for tagging if the shark was securely hooked (hook stuck firmly in jaw) and showed no visible signs of severe stress or health issues. Eligible sharks were secured in the water, alongside the boat, and then sexed, total length (TL) measured (to the nearest cm) and classified as immature or mature. Among female sharks, individuals without claspers, of TL ≥ 180 cm were considered sexually mature [56]. The maturity stage of male sharks, that is of individuals with claspers, was determined by assessing the degree of calcification of claspers [26, 27, 57].

Separate from the internal acoustic tag, an external marker tag (Rototag livestock tag, Destron Fearing or breeding pair ovine Allflex tag, Ritchie EID) was attached to the first dorsal fin to almost all acoustically tagged sharks. The tag was placed through a small hole made in the dorsal fin using a tag applicator. No external tag was attached to small (< 110 cm TL) sharks to avoid interference by the tag with dorsal fin growth. To facilitate quick tag implantation, relatively stress-free, tonic immobility was induced by inverting the shark [58] lengthways alongside the moving boat.

Uniquely coded acoustic tags (V9 for sharks < 110 cm TL, V16 for sharks > 110 cm TL [2], 69kHz, Innovasea Systems Inc., Canada) were surgically implanted between the skin and the muscle’s connective tissue which has an adhesive texture. In contrast insertion of tags into the coelomic cavity risks the tag moving internally and causing obstruction, as well as likely greater stress to the animal. An incision was made next to the body midline, anterior to the cloaca and a pocket, between the skin and the connective tissue, was created using a surgical scalpel. A sterilized tag (10% povidone iodine to reduce potential contamination of the surgical tag coating) was then inserted through the incision slit into the pocket. The incision slit in the skin was firmly closed with a series of braided-nylon sutures. Although some authors have reported external tags with an insertion point being lost [59], there was no evidence to suggest our tags were being ejected (for example we never observed continuous transmission to a single receiver from a non-moving tag). For release, the shark was turned back dorsal fin up, and the hook was removed from the mouth. Shark handling time was kept to a minimum to reduce the stress response in the animal [55, 60, 61], but typically took 15–20 mins from securing the shark to its release, given the need to strike a balance between operating quickly and taking care not to injure or alarm the animal more than necessary.

Tags emitted a unique acoustic code in intervals of approximately 90 s, with a delay that varied randomly in order to (1) reduce potential overlap of acoustic signals from other tags and, (2) maximize the monitoring duration over multiple years, considering the trade-off between transmission frequency and battery life expectancy (Innovasea Systems Inc., Canada). A tag’s expected battery life (tag life), based on information provided by the manufacturer ranged from 480–1915 days depending on tag type (V9, V16) and time since purchased. Expected tag life was noted for each tagged shark.

### Ethics statement

Capture, tagging and release of sharks was undertaken by a senior author (MG) holding a UK Home Office License to Undertake Experiments on Animals (License Number IFOE28429) in accordance with the terms of that license. The experimental protocol was approved by the Cayman Islands Department of the Environment and by the Cayman Islands Government Veterinary Service. To minimize stress to sharks while being tagged, sharks were caught with barbless hooks, the implanting of tags under the skin was undertaken with the sharks retained in the ocean alongside the boat (rather than being lifted from the water), prior to implantation a hypnotic state was induced in each shark by gently inverting it, sharks were tagged and released within 15–20 minutes of being captured, and any shark found to be immature or in less than good condition when captured was released without being tagged.

### Data collection

From 2010 to 2016 data (date and time (UTC), tag ID number) collected on receivers were downloaded annually by project members. Receivers were retrieved by SCUBA divers, the data downloaded, the batteries replaced, and the receivers then reattached to their mooring line. If the battery was still active at the time of the download, it was assumed that a receiver was working unless there was an error during the download. During the six-year period, minor array variations occurred due to receivers malfunctioning, unexplained disappearances, and loss due to environmental conditions. In 2016 a more serious issue was identified, resulting in modifications to the data collection protocol. Some receivers, although communicating (e.g. via LED flash sequence or Bluetooth) correctly, were not detecting and storing tag signals due to malfunctioning of the receiver software. This was not an obvious error and had to be checked at download for each receiver separately. Upon discovery, the protocol for receiver maintenance remained, but changed for data collection to reduce data loss. From 2017, data from receivers were collected quarterly, approximately every 3 months.

In addition to the outlined protocol, an activity check (placing the test tag next to the receiver before or during the download) was performed at each download. Confirmation of correct receiver function was given if the test tag was detected by the receiver. A malfunctioning receiver was replaced with another unit, when possible, or the receiver station was left without a receiver until the receiver was repaired and could be redeployed.

Data from receivers were downloaded with the VUE software (Innovasea Systems Inc., Canada), and the local date and time of the laptop corrected where time drift on the internal receiver clocks occurred. Data were stored in an Excel database.

### Data analysis

Analysis included the raw detection data from the study period, October 2010 (first tagged shark) to June 2019 (last download), from receiver stations that were equipped with functioning receivers for six or more months. Prior to analyses (as detailed in sections below), the raw detection data were standardised to reduce bias. Firstly, data were pre-processed by removing both single detections (likely to be errors [2]). Secondly, receivers were occasionally moved between receiver stations in the field. Both the receiver serial number and station name at each location were noted to ensure the correct location of a detected shark was reported. These were confirmed with a calendar representing a log of the location of each receiver serial number at any given point in time during the study period. Finally, the UTC date and time were adjusted to local time by subtracting 5 hours. Data pre-processing yielded 77,651 detections from 57 receivers (DoE array n = 47, CCMI array n = 10) of sharks tagged between 2010–2019.

Detection data from receivers were processed to define metrics detailed in the sections below. Continuous data were tested for normality and homogeneity of variance before hypothesis testing. None of the statistical analyses performed on the data met an assumption of normality using the Shapiro - Wilks normality test (α = 0.05), even after data transformation using log, square-root, and z score. Consequently, non-parametric tests were applied. The Levene’s test (α = 0.05) was used to test homogeneity; if this test was significant (p < 0.05), the assumption of homogeneity of variance of the data was not met and accounted for in the application of the subsequent statistical test. A p-value less than 0.05 (p < 0.05) was considered statistically significant, rejecting the null hypothesis (H_0_) and accepting the alternative hypothesis (H_1_). The exact p-value was reported for values greater than 0.001 otherwise ‘p < 0.001’ was reported. All statistical analyses were conducted in RStudio (R v3.6.1) using packages ‘car’, ‘dunn.test’, ‘vegan’ and ‘igraph’. Graphs were drawn in Microsoft Excel (v1911), networks were visualized in RStudio and maps were plotted using ESRI’s ArcGIS Desktop v10.4.

#### Tagging effort assessment

A Chi-squared goodness of fit test was used to test the null hypotheses that the number of tagged sharks in the sample were not significantly different between islands, sexes, or maturity. Data were processed to define the number of detections, the number of detection days (number of days of two or more detections of a shark), and the detection period (number of days between the date of tagging and the date of a shark’s last detection). To test for significant differences between sexes or maturity, a two-way non-parametric Mann-Whitney U test was performed, and for significant differences between capture islands, a two-way non-parametric Kruskal-Wallis rank sum test.

#### Residency

The extensive use of acoustic telemetry and typically large data sets have resulted in a wide range of analyses being used to assess movements of tagged sharks, including Residency Index (RI), Site-Fidelity Index (SFI), minimum linear displacement (MLD) and network analysis [2, 11, 12, 42, 62–64]. The terms ‘residency’ and ‘site-fidelity’ are used interchangeably in the literature referring to either the study area [11, 49, 65, 66] and/or specific sites [2, 25, 67]. To avoid confusion, in this study residency was defined as the presence within the entire acoustic array, and site-fidelity was the attachment to a specific receiver station.

Residency of sharks to the Cayman Islands was assessed using two different approaches: (1) a standardized Residency Index (RI) based on detection days and (2) the Classification of Residency (CR) based on behaviour patterns. For each shark, the RI (derived from [2, 62, 68]) was calculated (Eq 1) from the total number of days each shark was present (≥ two detections on the same day, [62, 68]) within the entire acoustic array (= number of detection days) over the maximum number of days the shark could possibly have been detected (= tag life) assuming the tag was active for the period of estimated battery life [2, 63, 64]. For sharks tagged in 2019, tag life would exceed the final date of the study. For these sharks, the RI was defined as the number of detection days divided by the number of days from the tagging date to the last date of the study period. The RI ranges from 0–1, indicating low to high residency to the receiver array. Sharks that were detected every single day, from date of tagging to last date of tag life, would have a RI of 1.


$$RI=\frac{number\;of\;detection\;days}{tag\;life\;(days)}$$ (1)

To determine the overall residency of sharks, the mean (± SE) RI and its range were reported. The monthly presence (months with ≥ one detection day) and the individual monitoring period (i.e. tag life) of sharks were plotted on a timeline for visual interpretation. To test the null hypothesis that the RI was not significantly different between sexes or maturity, a two-way non-parametric Mann-Whitney U test was performed, and for significant differences between capture island, a two-way non-parametric Kruskal-Wallis rank sum test.

The Classification of Residency (CR) approach categorized sharks based on their behavioural pattern (derived from Werry et al. [48]) into four categories: Passer-by, Transient, Pseudo-resident and Resident (Table 1). For each shark, data were processed to define the number of detection days, the detection period (number of days from date of tagging to date of last detection), and the number of detection months (number of months with ≥ one detection day anywhere within the acoustic array). The percentage of a shark’s presence during its detection period was calculated by dividing the number of detection days by the detection period then multiplied by 100 and used to classify individuals based on criteria outlined in Table 1.

**Table 1:** Classification criteria, derived from Werry et al. [48] of localized behaviour patterns exhibited by acoustically tagged Caribbean reef sharks in the Cayman Islands (2010–2019).

Classification	Criteria
Passer-by	Individuals that were never detected after the first 30 days (‘Month’) of detections post tagging.
Transient	Individuals that were re-detected after a period greater than a Month of no detections, but were detected for < three detection Months in total.
Pseudo-resident	Individuals that were detected for three Months or more and for < 30 % of their potential detection period.
Resident	Individuals that were detected for three Months or more and for > 30 % of their potential detection period.

Proportions of Passer-by, Transient, Pseudo-resident and Resident sharks were reported and only sharks classified as Pseudo-resident and Resident, jointly referred to as ‘*resident’*, were further analysed. To test the null hypothesis that residency differed significantly among sex or maturity, number of resident sharks were compared using a Chi-squared goodness of fit test. Significant differences in numbers of *resident* sharks between capture islands were determined using a Fisher exact probability test, allowing for different sample effort [69].

#### Site-fidelity

Site-fidelity of individual sharks was examined by (1) using a standardised Site-Fidelity Index (SFI) to identify the ‘primary site’ (most frequently utilized receiver, [11, 70, 71]) of a shark, and (2) investigating the relationship between minimum linear displacement (MLD) and ranked receiver sites (derived from [2]). For each shark, data on receivers that detected the shark were processed to define the number of detections, the number of detection days (≥ two detections), and to calculate the MLD between its primary and all other receivers that detected the shark. Assuming a shark was on the peripheral edge of each receiver’s detection radius [11], the position data of the two receivers were converted into distance travelled, using the great-circle distance formula (https://www.movable-type.co.uk/scripts/latlong.html), measured in kilometres (km), adding 0.6 km (detection radius of a receiver (0.3 km) x 2).

To calculate the SFI, a Detection Index (DI) was used, aiming to reduce bias in cases where the size of the home range or the behaviour of a shark resulted in a reduced / increased number of detections at a site. A shark may not be detected for the entire time that it spends closest to its primary receiver site because it may nevertheless be outside the detection range (300 m) of that receiver for much of the time, but detections should nevertheless be relatively constant over an extended period of time (i.e. over many detection days). Indeed, a shark may for a period of time (e.g. due to feeding behaviour or environmental conditions) be within the detection range of an adjacent site, rather than its primary one.

For each shark, a DI for those receivers that detected the shark was calculated by multiplying the proportion of detections (number of detections on receiver / total number of detections of shark) and the proportion of detection days (number of detections days on receiver / total number of detection days of shark). Using the DI of receivers, an SFI was calculated (Eq 2), ranging from 0–100%. For each shark, the SFI was used to rank the receivers that detected the shark. The receiver with the highest SFI was assigned the 1° receiver site and the receiver with the lowest SFI the largest rank number.


$$SFI\;(\%)=\frac{Detection\;Index\;of\;receiver}{sum\;of\;Detection\;Indices\;from\;all\;receivers\;that\;detected\;the\;shark\;}\;x\;100$$ (2)

The SFI values were pooled and tested for significant differences between ranks using a two-way non-parametric Kruskal-Wallis rank sum test and significant differences between ranks were determined using a post-hoc Dunn test. The SFI of receivers was plotted against the MLD and the relationship assessed using a non-parametric Spearman’s rank correlation [2]. If site-fidelity was exhibited by sharks, the SFI of primary receivers was expected to be significantly higher compared to that of the other ranks and MLD was expected to increase with decreasing SFI [2].

To test whether the SFI was significantly correlated to shark size (TL) a non-parametric Spearman’s rank correlation was used, and to test for significant differences between sexes a two-way non-parametric Mann-Whitney U test. If the degree of site-fidelity was independent of shark size and females were exhibiting higher site-fidelity than males, the SFI was expected not to change with increasing shark size and to be significantly higher for females than for males.

#### Coastal space use

The use of coastal space by sharks was assessed using three approaches: (1) the utilization of the receiver array using a Utilization Index (UI), (2) home range and long-distance movements using the minimum linear displacement (MLD), and (3) the analysis of demographic-, diel- and season-specific movements using network analysis, an Activity Index (AI) and detection patterns.

*Utilization of receiver array*. The utilization of receivers by sharks was analysed using a Utilization Index (UI) in order to account for spatiotemporal variations in the array design and receiver function. The use of the UI was intended to offer a realistic representation of the actual receiver use by accounting for both the amount of time sharks spend at a receiver (detection days) and the number of sharks detected. This reduces the risk of over-weighting detections from receivers that were occupied by one shark for a long time (multiple detection days but from only one individual) in comparison with receivers that were used by many individuals, none of which appeared to stay for long (i.e. less detection days but from multiple individuals).

For each receiver station, the number of working days was defined as the number of days the station was equipped with a functioning receiver during the study period and this number was used to calculate ‘Station Days’ (number of detection days / number of working days). The UI for each receiver station was calculated (Eq 3) and mapped for visual identification of areas with relative high shark occurrence (‘hotspots’).


$$UI=\frac{number\;of\;shark\;detected}{total\;no.\;of\;sharks\;detected\;in\;the\;array\;}\;x\;\frac{Station\;Days}{total\;no.\;of\;detection\;days\;in\;the\;array}$$ (3)

*Home range and long-distance movements*. For the analysis of linear home range size, potential long-distance movements (> 50 km: [11, 23]) and movements between the Cayman Islands, the individual MLD was calculated and the number of islands that each shark was detected on defined. It was considered that the shark had moved between islands if it was detected on more than one island. The MLD of each shark was defined as the linear distance between the two most distant receivers which detected the shark [11], as described above. If a shark was only detected on one receiver after tagging, the distance between tagging location and receiver was calculated and only the detection radius of one receiver (0.3 km) added. To determine whether the MLD was significantly correlated to shark size (TL), a non-parametric Spearman’s rank correlation was used, and to test for significant differences between sexes or maturity, a two-way non-parametric Mann-Whitney U test.

*Movements based on sex*, *maturity stage*, *season*, *and day period*. To determine whether coastal space use differed significantly between sexes, maturity, season (summer: April–September, winter: October–March), or diel period (day: 06:30–18:29, night: 18:30–06:29), network analysis was applied. Coastal space use was compared using three approaches: (1) visual comparison of networks, (2) testing two group-specific matrices for significant correlation (‘overlap’) using a Mantel test [72–74], and (3) testing the node degree for significant differences between sexes, maturity, season, or diel period using a two-way non-parametric Mann-Whitney U test [49]. Additionally, for each sex, maturity, season and diel period, the number of receivers utilised, and total detections were reported. Only two seasons were defined because classification into the more usual four seasons was inappropriate for the tropical Cayman marine environment which changes little throughout the year.

Prior to analysis, the data were processed into two files: (1) a receiver dataset, containing the receiver location, serial number, and receiver station name; (2) a detection dataset that consisted of the raw detections together with shark characteristics (sex, maturity) and factors of interest (season, diel period). Subsequently, adjacency matrices for subsets (depending on analysis) were defined where edges represent directed movements (in- and outgoing) between nodes (pairs of receivers) [72] using the igraph R package [75] following Whoriskey et al. [74]. Successional detections between nodes were treated as a connection between those nodes [13]. The node degree quantified the number of direct connections (in- and out-going) a receiver had with any other receiver in the array [13, 74], defining the amount of traffic through a receiver [13]. The edge weight comprised of the number of times a movement was made between two nodes, a metric for mobility of individuals within the network [74]. Group-specific networks were visualized as nodes, showing the position of receivers, their node degree and the weighted edges connecting them.

Monthly activity of sharks was assessed using monthly detection patterns and an Activity Index (AI). For sharks with a detection period of ≥ one year and/or that were detected on ≥ two islands, the monthly detection pattern (month with ≥ detection day) was plotted across months and years for visual interpretation. Across all years and sharks, a monthly AI was calculated by multiplying the proportion of detection days (number of a total detections days in each month / number of total working days of all receiver stations in each month) with the proportion of detected sharks (number of sharks detected in each month / total number of detected sharks in that month across years), and this monthly AI plotted against months for visual interpretation. It was hypothesised that the relative AI and monthly detections increased during summer (April–September).

## Results

### Tagging effort assessment

Over the nine-year study period, a total of 66 Caribbean reef sharks were tagged during periodic tagging expeditions. Not all sharks were tracked simultaneously, but the tag battery life of some individual sharks covered the entire study period. Shark presence was highly variable from year to year and between individuals with a minimum of four and maximum of 17 sharks being monitored at any one time (S3 Fig). Sharks were captured around all three Cayman Islands but significantly more (Chi-squared test: χ^2^ = 23.545, df = 2, p < 0.001) sharks were tagged on Little Cayman (60%) than on Grand Cayman (26%) or Cayman Brac (14%). Sharks ranged from 76–196 cm TL (mean = 149 cm ± 6.47 (SE), Table 2). The numbers of tagged sharks did not differ significantly between sexes (Chi-squared test: χ^2^ = 0.970, df = 1, p = 0.325) or with maturity (Chi-squared test: χ^2^ = 0.546, df = 1, p = 0.460).

**Table 2:** Summary of tagging and acoustic data for Caribbean reef sharks (n = 39) detected in the Cayman Islands during 2010–2019 and used for data analysis.

Tag ID	Sex	Mat. stage	TL	Tagging		Tag life	Det. period	No. of			RI	CR	MLD	No. of utilized		SFI
				Year	Isl.			Dets	days	months				Recs	Isl.	
48033	F	M	196	2010	GC	1167	15	83	15	1	0.0129	Pass.-by	32.7	7	1	74
48035	F	M	192	2010	LC	1167	4	4	1	1	0.0009	Pass.-by	114.5	1	1	100
48039	F	IM	159	2010	LC	1167	1,159	22	15	5	0.0129	Ps.-res.	7.1	1	1	100
48042	F	IM	138	2010	LC	1167	6	54	5	1	0.0043	Pass.-by	18.4	3	1	54
32353	M	M	156	2011	CB	1262	1,179	2,094	254	25	0.2013	Ps.-res.	22.3	5	1	70
32354	F	IM	170	2011	CB	1262	1,063	24,220	232	18	0.1838	Ps.-res.	20.0	4	1	100
32355	M	M	171	2011	CB	1262	97	82	9	3	0.0071	Ps.-res.	142.2	3	3	99
32357	F	IM	133	2011	CB	1262	8	402	6	1	0.0048	Pass.-by	4.3	1	1	100
32360	F	IM	175	2011	LC	1262	78	112	13	2	0.0103	Trans.	28.9	5	2	59
48017	M	IM	142	2011	LC	1167	12	44	7	1	0.0060	Pass.-by	18.6	3	2	67
48020	M	M	159	2011	GC	1167	86	53	8	2	0.0069	Trans.	0.8	1	1	100
28955	F	M	195	2012	GC	1237	1,037	4,206	595	35	0.4810	Resident	148.2	11	2	100
32367	F	M	190	2012	LC	1262	6	3	1	1	0.0008	Pass.-by	2.2	1	1	100
32361	M	M	159	2013	LC	1262	1,598	1,711	172	32	0.1363	Ps.-res.	29.9	20	2	96
32364	M	M	160	2013	LC	1262	429	3,916	151	13	0.1197	Resident	42.4	9	2	80
32366	F	IM	103	2013	LC	1262	691	1,249	114	20	0.0903	Ps.-res.	3.6	2	1	99
32369	M	M	169	2013	LC	1262	1	3	1	1	0.0008	Pass.-by	0.9	1	1	100
32352	M	IM	120	2015	GC	1262	14	23	5	2	0.0040	Pass.-by	6.0	1	1	100
32359	F	M	191	2015	GC	1262	13	5	3	1	0.0024	Pass.-by	12.9	1	1	100
22285	F	IM	132	2016	LC	1915	10	683	7	1	0.0037	Pass.-by	8.2	2	1	75
22291	M	M	156	2016	LC	1915	539	17,088	544	20	0.2841	Resident	19.7	20	1	100
22292	M	M	172	2016	LC	1915	24	44	9	1	0.0047	Pass.-by	8.6	4	1	71
22295	M	M	152	2016	LC	1915	15	2,899	15	1	0.0078	Pass.-by	0.9	2	1	100
22296	F	M	181	2016	GC	1915	3	59	2	1	0.0010	Pass.-by	1.2	1	1	100
32371	F	IM	169	2016	LC	1262	18	223	17	1	0.0135	Pass.-by	4.0	4	1	80
43700	M	IM	94	2016	LC	481	5	57	3	1	0.0062	Pass.-by	8.2	3	1	56
43701	M	IM	98	2016	LC	481	546	3,415	200	18	0.4158	Resident	17.0	16	1	82
43702	M	IM	83	2016	LC	481	5	22	2	1	0.0042	Pass.-by	8.2	2	1	59
45050	M	IM	76	2016	LC	480	1	21	1	1	0.0021	Pass.-by	1.9	1	1	100
45051	M	IM	102	2016	LC	480	6	201	6	1	0.0125	Pass.-by	1.0	2	1	96
43700	M	IM	94	2016	LC	481	5	57	3	1	0.0062	Pass.-by	8.2	3	1	56
43701	M	IM	98	2016	LC	481	546	3,415	200	18	0.4158	Resident	17.0	16	1	82
43702	M	IM	83	2016	LC	481	5	22	2	1	0.0042	Pass.-by	8.2	2	1	59
45050	M	IM	76	2016	LC	480	1	21	1	1	0.0021	Pass.-by	1.9	1	1	100
45051	M	IM	102	2016	LC	480	6	201	6	1	0.0125	Pass.-by	1.0	2	1	96
22301	M	M	167	2017	LC	1915	88	4	1	2	0.0005	Trans.	39.4	1	1	100
22305	M	M	183	2017	LC	1915	282	6,915	225	11	0.1175	Resident	16.3	8	1	40
43695	F	IM	83	2017	LC	481	4	126	4	1	0.0083	Pass.-by	1.9	5	1	85
22315	M	M	152	2019	LC	1915	8	698	8	2	0.0042	Pass.-by	2.3	2	1	100
22316	M	M	170	2019	LC	1915	13	1,059	12	2	0.0063	Pass.-by	7.4	5	1	77
22324	F	M	190	2019	GC	1915	16	2,037	15	1	0.0078	Pass.-by	3.8	2	1	100
22325	M	M	185	2019	GC	1915	28	2,229	26	2	0.0136	Pass.-by	3.8	2	1	99
45052	F	IM	89	2019	LC	480	66	525	34	4	0.0708	Resident	10.4	5	1	97
45053	M	IM	105	2019	LC	480	62	1,060	47	3	0.0979	Resident	4.0	3	1	52

Tag ID = Tag Identification number, F = female, M = male, Mat. stage = Maturity stage: IM = immature, M = mature; Isl. = island: GC = Grand Cayman, LC = Little Cayman, CB = Cayman Brac; RI = Residency Index; CR = Classification of Residency: Pass.-by = Passer-by, Trans. = Transient, Ps.-res. = Pseudo-resident; MLD = Minimum linear displacement; SFI = Site-Fidelity Index of primary receiver. Units: TL = Total Length cm, Tag life/Detection period = days, MLD = km, SFI = %

Of the 66 tagged sharks, 39 (59.1%) individuals (male = 22, female = 17, immature = 18, mature = 21) were detected on the array, generating a total of 77,651 detections (Grand Cayman = 11%, Little Cayman = 54%, Cayman Brac = 35%) over 3,816 detections days (Table 2). Tagged individuals were detected for overall detection periods of 0–1,598 days (mean = 139.9 days ± 42.01 (SE)) with a mean of 42.2 ± 13.77 (SE) detection days per shark (range = 0–595 days), and a mean of 1,176.49 ± 460.58 (SE) detections per shark (range = 0–24,222). Detection metrics did not differ significantly between sex, maturity, or capture island (statistical results are reported in S4 Fig).

### Residency

**Fig 2:**
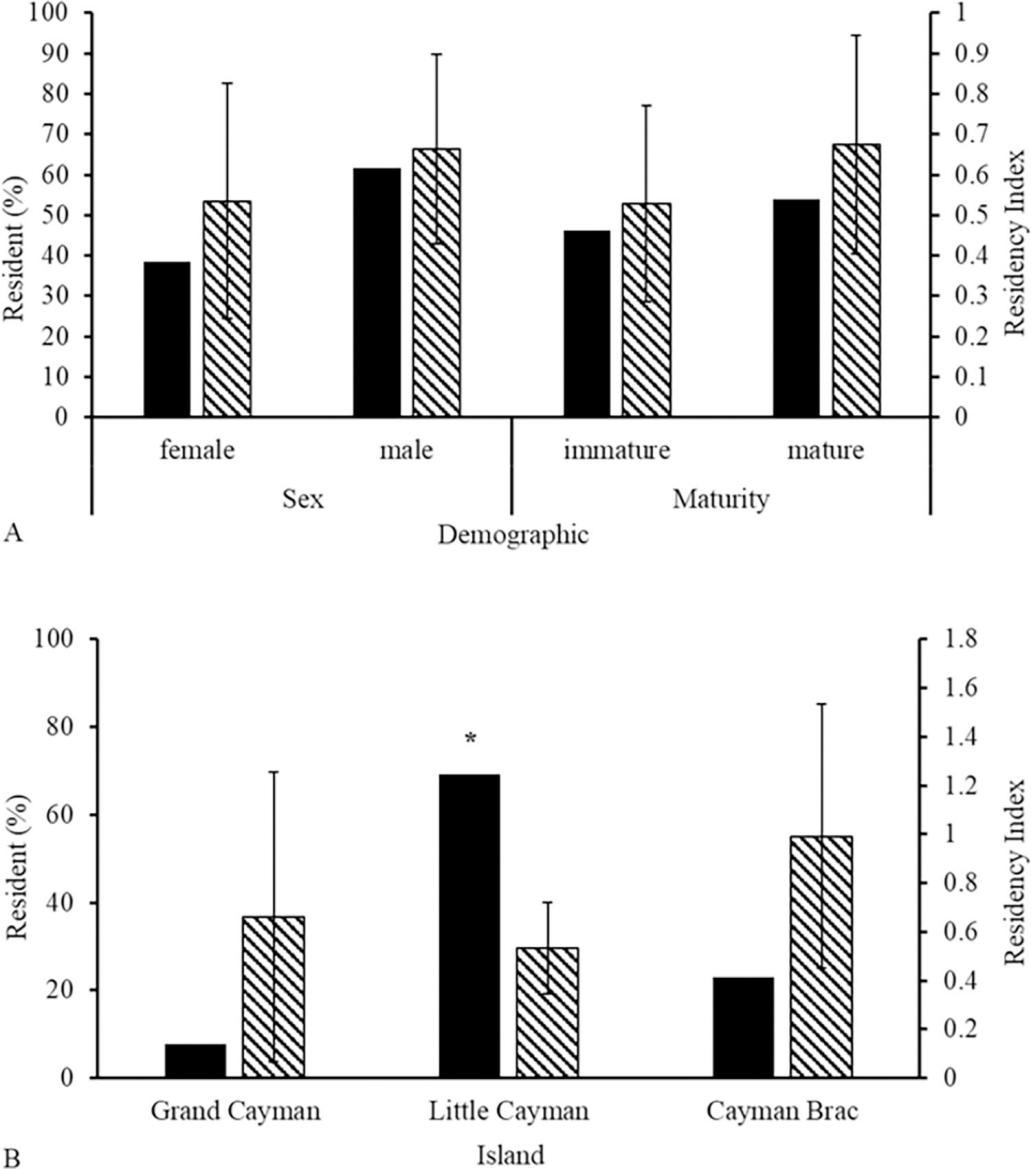
Comparison of percentage of resident (Pseudo-resident and Resident) (CR) sharks (black bars) and mean (± SE) Residency Index (RI) of sharks (striped bars) (n = 39) between A) demographics and B) capture island. Significant difference (p < 0.05) is marked with *.

Tagged sharks were detected for a mean of 6% of their monitoring period (RI mean ± SE = 0.06 ± 0.02, range = 0.0005–0.48). Only 13 individuals (CR: Resident and Pseudo-resident sharks = 19%) showed evidence of resident behaviour, being detected throughout the year and in some cases in successive years. The majority were detected sporadically (Transient = 4.5%), or for only up to 30 days (Passer-by = 35%) post-tagging (Table 2), regardless of sex (RI: Mann-Whitney U test: W = 176, p = 0.766; CR: Chi-squared test: χ^2^ = 0.69231, df = 1, p = 0.405) or maturity (RI: Mann-Whitney U test: W = 203.5, p = 0.693; CR: Chi-squared test: χ^2^ = 0.077, df = 1, p = 0.782, Fig 2A). Residency of sharks differed between capture island with a significant difference in CR (Chi-squared test: χ^2^ = 12, df = 2, p = 0.003) but not in RI (Kruskal-Wallis rank sum test: χ^2^ = 1.243, df = 2, p = 0.537). Overall, residency of sharks tagged on Little Cayman was greater with relatively little variation, followed by Cayman Brac and Grand Cayman, both of which had greater variation in individual residency (Fig 2B).

### Site-fidelity

**Fig 3:**
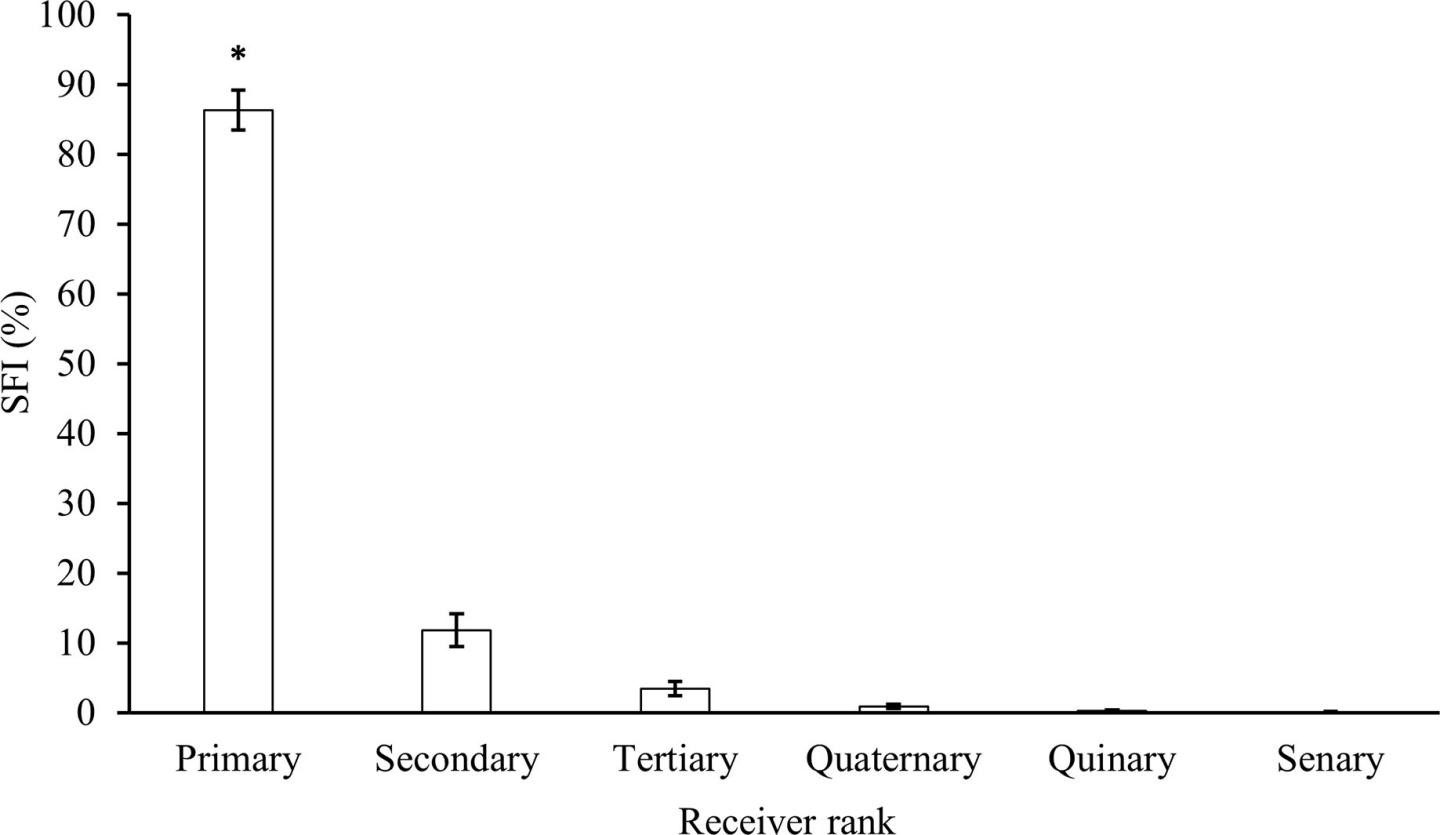
Mean (± SE) Site-Fidelity Index (SFI) of ranked receivers from Caribbean reef sharks (n = 39) in the Cayman Islands between 2010–2019. Significant differences are marked with * and post-hoc Dunn test results reported in S1 Table.

**Fig 4:**
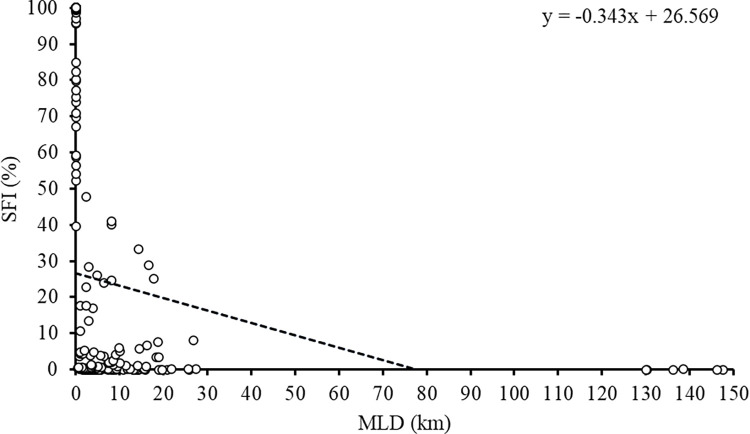
The Site-Fidelity Index (SFI) of receivers against the minimum linear displacement (MLD) between a shark’s primary receiver and another for Caribbean reef sharks in the Cayman Islands (n = 39; 2010–2019). A significant Spearman correlation is shown including regression line and equation.

Particular sharks were detected on up to 20 different receivers (mean for all individuals = 4.36 receivers ± 0.78 SE), but sharks were detected significantly more (Kruskal-Wallis rank sum test: χ^2^ = 135.62, df = 19, p < 0.001; post-hoc Dunn test S1 Table) at their primary receiver site than over the rest of the array and then mainly at neighbouring receiver sites (Fig 3). The Site-Fidelity Index (SFI) and minimum linear displacement (MLD) were significantly negatively correlated (Spearman rank correlation: S = 1302999, rho = -0.591, p < 0.001), SFI of receivers decreased with increasing MLD between the receiver and the primary receiver site (Fig 4), indicating that sharks tended to show significant site-fidelity to their primary receiver site.

Site-fidelity did not differ between sex (Mann-Whitney U test: W = 229, p = 0.235; S5A Fig) nor was there a significant correlation between SFI and TL (Spearman rank correlation: S = 7787.5, rho = 0.212, p = 0.196; S5B Fig). However, it is noteworthy, that females and larger sharks appeared to exhibit higher site-fidelity to their primary receiver than males and smaller individuals, respectively.

### Coastal space use

#### Utilization of receiver array

**Fig 5:**
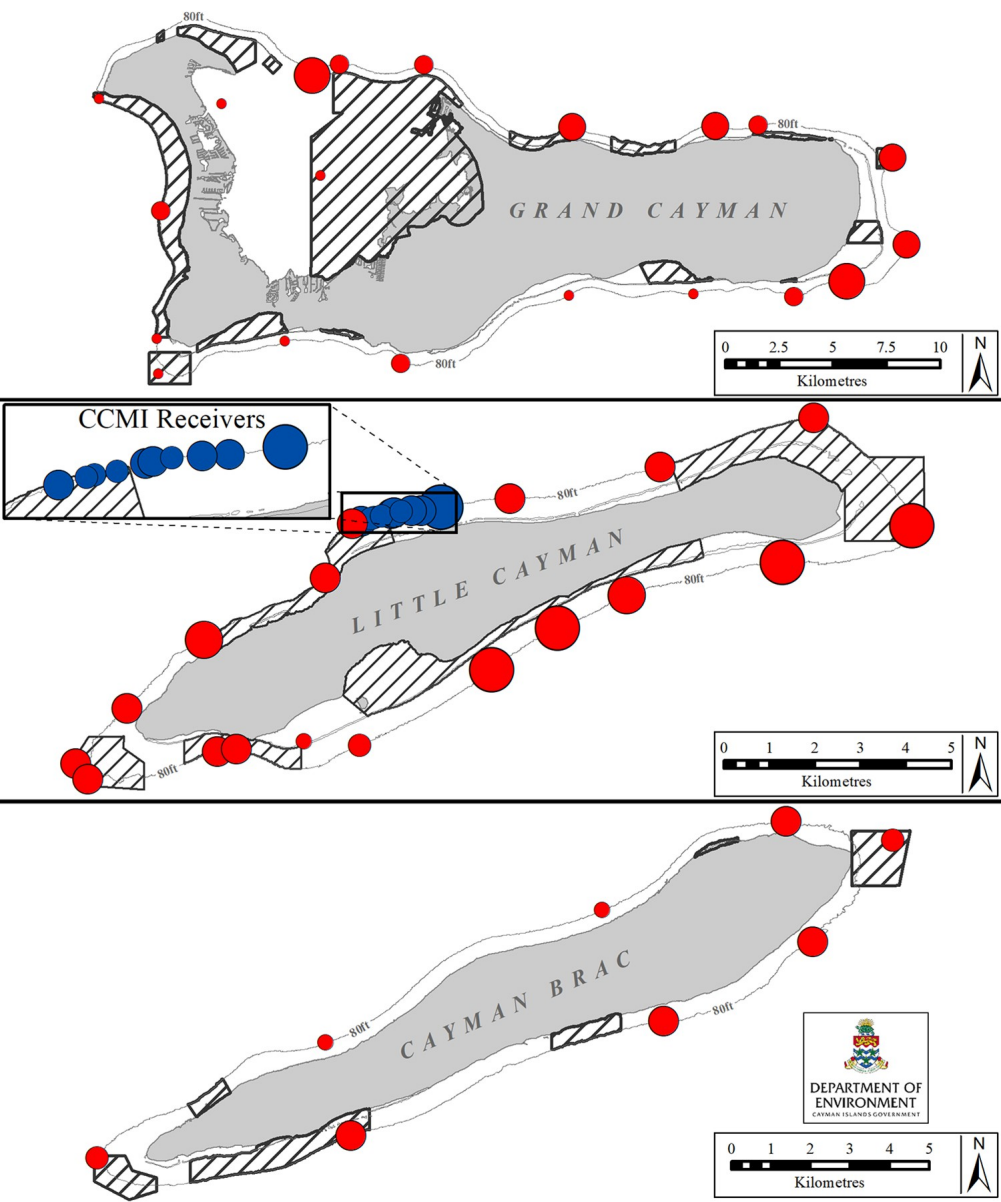
The coastal space use of Caribbean reef sharks (n = 39) in the Cayman Islands (2010–2019). Circles indicate receiver stations and the circle area is scaled by the Utilization Index (UI = proportion of detected sharks times the proportion of station days). Receivers with greater UI are indicated by larger circles than receivers with smaller UI. Black line around each island indicates the 25 m depth contour and shaded areas represent MPAs. Created by the Department of the Environment, Cayman Islands Government.

The Utilization Index (UI = proportion of detected sharks multiplied by the proportion of station days) appeared to be greater at receivers on the North and SE sides of Grand Cayman, the South side, NW, and North sides of Little Cayman, and the E and the South side of Cayman Brac than at other sites of the array (Fig 5).

#### Home range and long-distance movements

**Fig 6:**
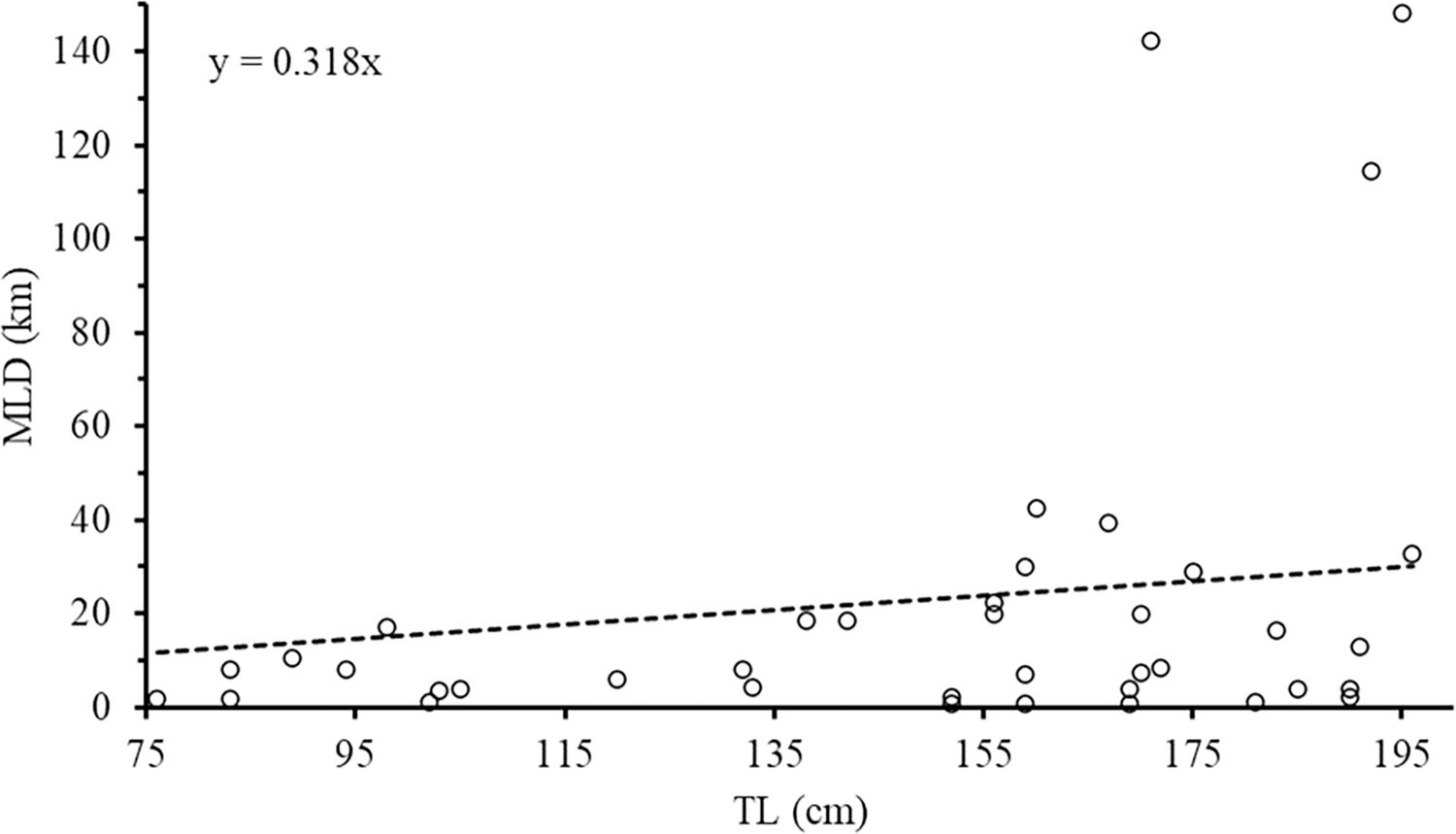
The minimum linear displacement (MLD) against the TL of Caribbean reef sharks (n = 39), including regression-line and equation.

Overall, the linear home range (MLD) of sharks was 21.13 km ± 5.65 (SE), although most individuals (56%) had an observed linear home range of < 10 km (Table 2), which was not significantly different between sexes (Mann-Whitney U test: W = 199.5, p = 0.734) or maturity (Mann-Whitney U test: W = 157, p = 0.375; S6 Fig). Six sharks were detected on more than one island and three sharks undertook long-distance movements (> 105 km) being detected on both Grand Cayman and the Sister Islands more than 100 km apart (Table 2). The maximum distance recorded was 148.21 km across deep (up to 2,000 m depth) open ocean (Table 2). There was a significant positive correlation between MLD and TL of sharks, indicating that larger sharks tended to move greater distances than smaller individuals (Spearman correlation: S = 6737.1, rho = 0.318, p = 0.048, Fig 6).

#### Sex- and maturity stage-specific movements

**Fig 7:**
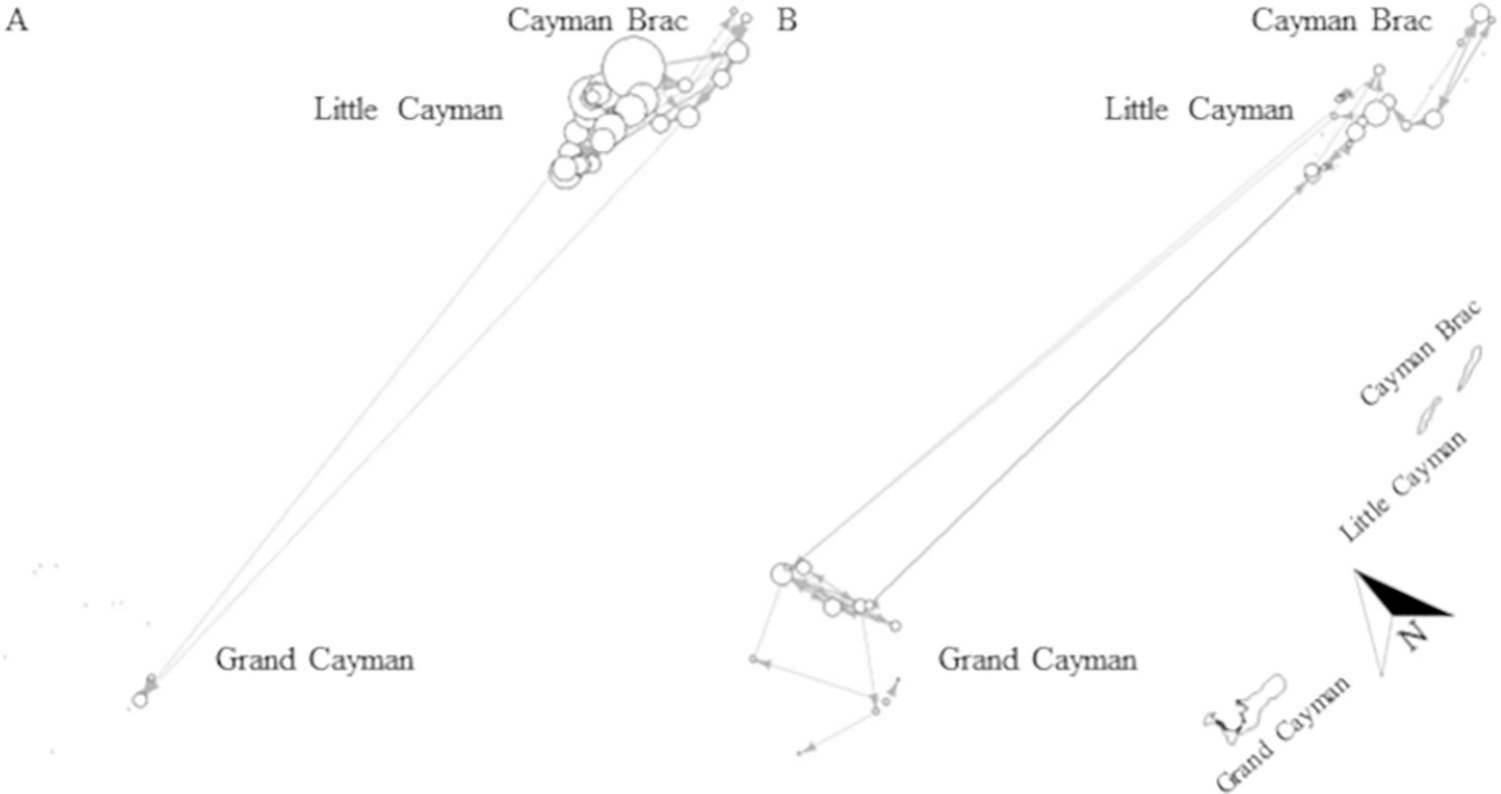
Sex-specific network for A) male and B) female Caribbean reef sharks across the Cayman Islands. Black dots (no connections) or circles (connections) indicate receiver stations and the circle size is scaled by node degree (i.e. receivers with more connections are indicated by larger circles than receivers with fewer/no connections); arrows indicate the directions of movement between receiver stations and the line thickness is scaled by the number of movements between two receivers. Male node degrees ranged from 1–17 connections; male edges ranged from 1–14 movements, female node degrees ranged from 1–7 connections; female edges ranged from 1–8 movements. Networks were created in R-studio. Note: this graph is not spatially oriented, but a map of the islands is shown for reference at the bottom right corner. Map was created by the Department of the Environment, Cayman Islands Government.

Overall, there was evidence of sex-based spatial segregation. Although sexes generated similar number of detections (males: 56%, females: 44%) and were detected on similar number of receivers (males: 38 receivers, females: 34 receivers), network analysis showed only weak overlap in coastal space use (Mantel test: r = 0.245; p < 0.001, Fig 7), with the majority of females being detected on different receivers than those of males (Grand Cayman and Cayman Brac) and fewer females than males being detected on receivers that detected both sexes. Males were significantly more mobile than females (Mann-Whitney U test: W = 660.5, p < 0.001), generally moving more between receivers (Fig 7A). While it appears that some individuals of both sexes moved between all three islands, male sharks showed more connections between the two Sister Islands while females showed more connections between Grand Cayman and the Sister Islands (Fig 7B).

**Fig 8:**
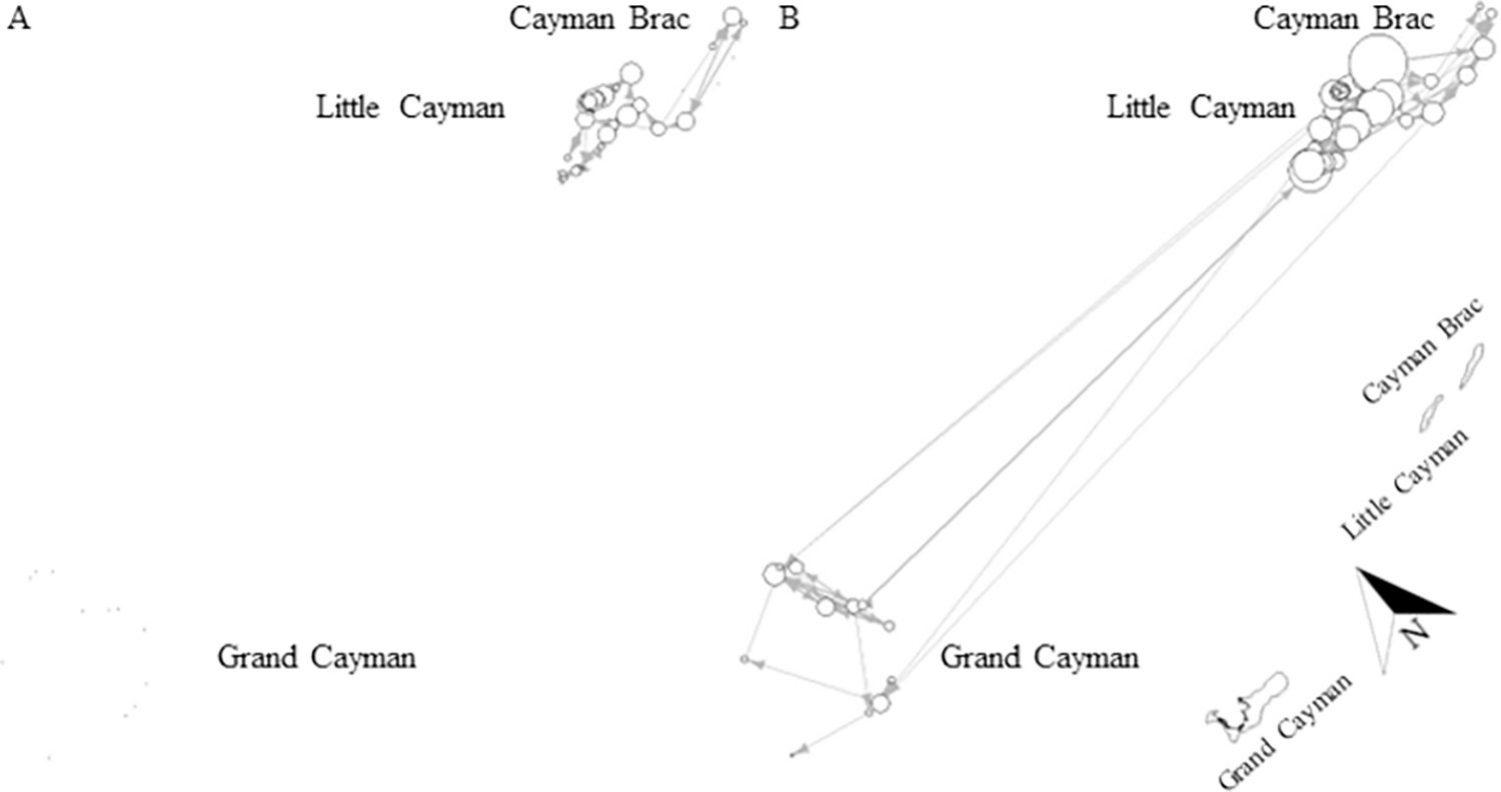
Maturity-specific network for A) immature and B) mature Caribbean reef sharks across the Cayman Islands. Black dots (no connections) or circles (connections) indicate receiver stations and the circle size is scaled by node degree (i.e. receivers with more connections are indicated by larger circles than receivers with fewer/no connections); arrows indicate the directions of movement between receiver stations and the line thickness is scaled by the number of movements between two receivers. Immature node degrees ranged from 1–6 connections; immature edges ranged from 1–8 movements, mature node degrees ranged from 1–16 connections; mature edges ranged from 1–13 movements. Networks were created in R-studio. Note: this graph is not spatially oriented, but a map of the islands is shown for reference at the bottom right corner. Map was created by the Department of the Environment, Cayman Islands Government.

There was a difference in extent of movements between maturity stages, network analysis showing only a minor overlap in use of coastal space (Mantle test: r = 0.3677, p < 0.001, Fig 8). Generally, mature individuals appeared significantly more mobile than immature sharks (Mann-Whitney U test: W = 628.5, p < 0.001, Fig 8), generated more detections (immature: 42%, mature: 58%) and were detected on more receivers (immature: 31 receivers, mature: 47 receivers) within the array. However, this finding could be an artefact due to the difference in transmission range of V16 compared to V9 tags (used on very small individuals). It is worth noting that immature sharks are not shown on Grand Cayman (Fig 8A) because even though immature individuals were tagged and detected, they did not move between receivers (i.e. node degree = 0). Upon visual comparison of networks, it appears that both younger and older sharks moved between islands, however, all long-distance movements (between Grand Cayman and Sister Islands, > 100 km) were undertaken by mature individuals (Fig 8B), whereas immature sharks moved only between Little Cayman and Cayman Brac (Fig 8A). It is also worth mentioning that often mature sharks appeared to move between receivers that were not adjacent to each other, including movements between receivers on different islands, while immature sharks seemed to follow a path connecting receivers adjacent to each other, even when moving between the Sister Islands (Fig 8).

#### Seasonal movements

**Fig 9:**
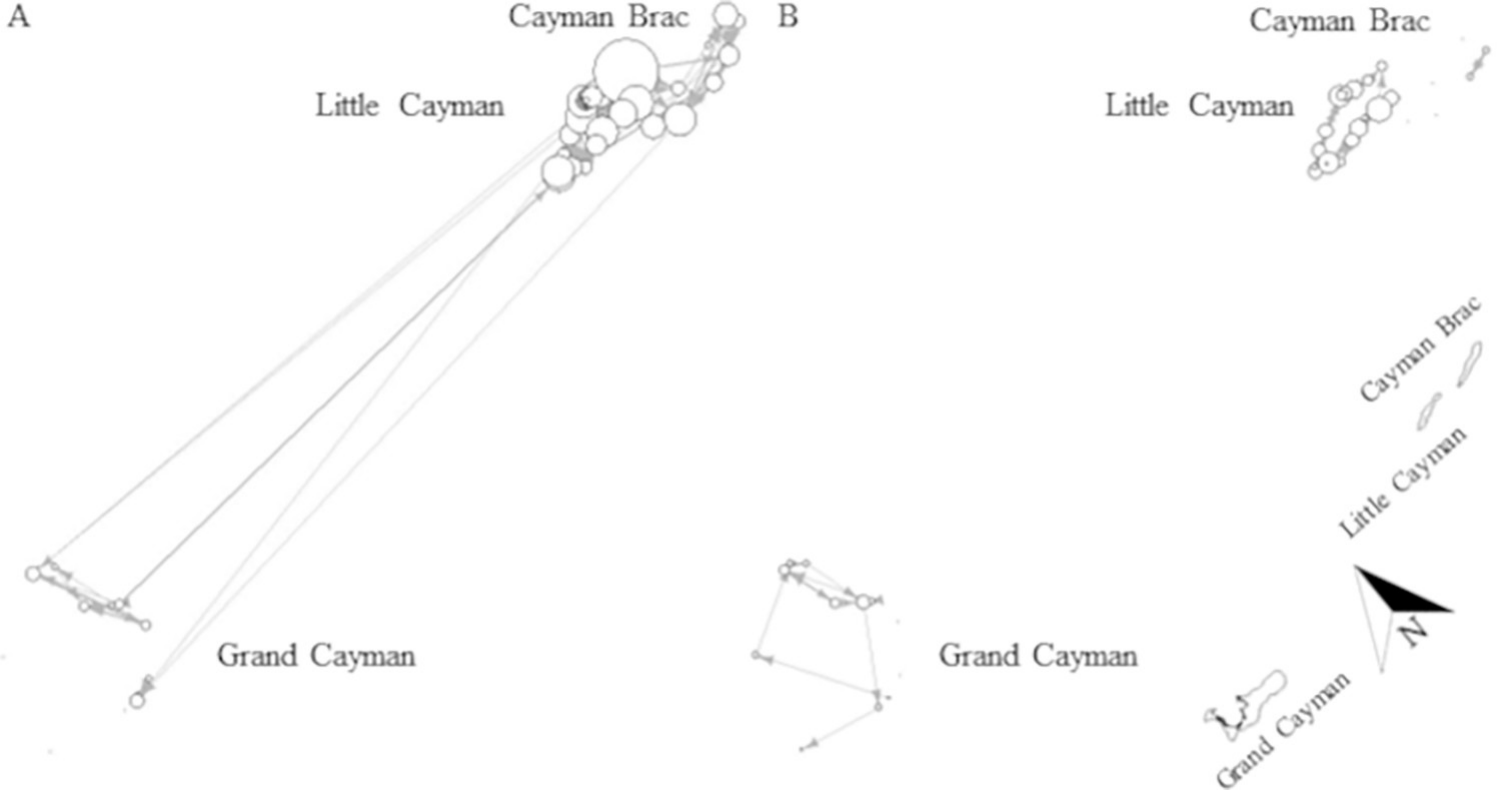
Networks Caribbean reef shark behaviour in A) summer and B) winter across the Cayman Islands. Black dots (no connections) or circles (connections) indicate receiver stations and the circle size is scaled by node degree (i.e. receivers with more connections are indicated by larger circles than receivers with fewer/no connections); arrows indicate the directions of movement between receiver stations and the line thickness is scaled by the number of movements between two receivers. Summer node degrees ranged from 1–18 connections; summer edges ranged from 1–15 movements, winter node degrees ranged from 1–7 connections; winter edges ranged from 1–6 movements. Networks were created in R-studio. Note: this graph is not spatially oriented, but a map of the islands is shown for reference at the bottom right corner. Map was created by the Department of the Environment, Cayman Islands Government.

**Fig 10:**
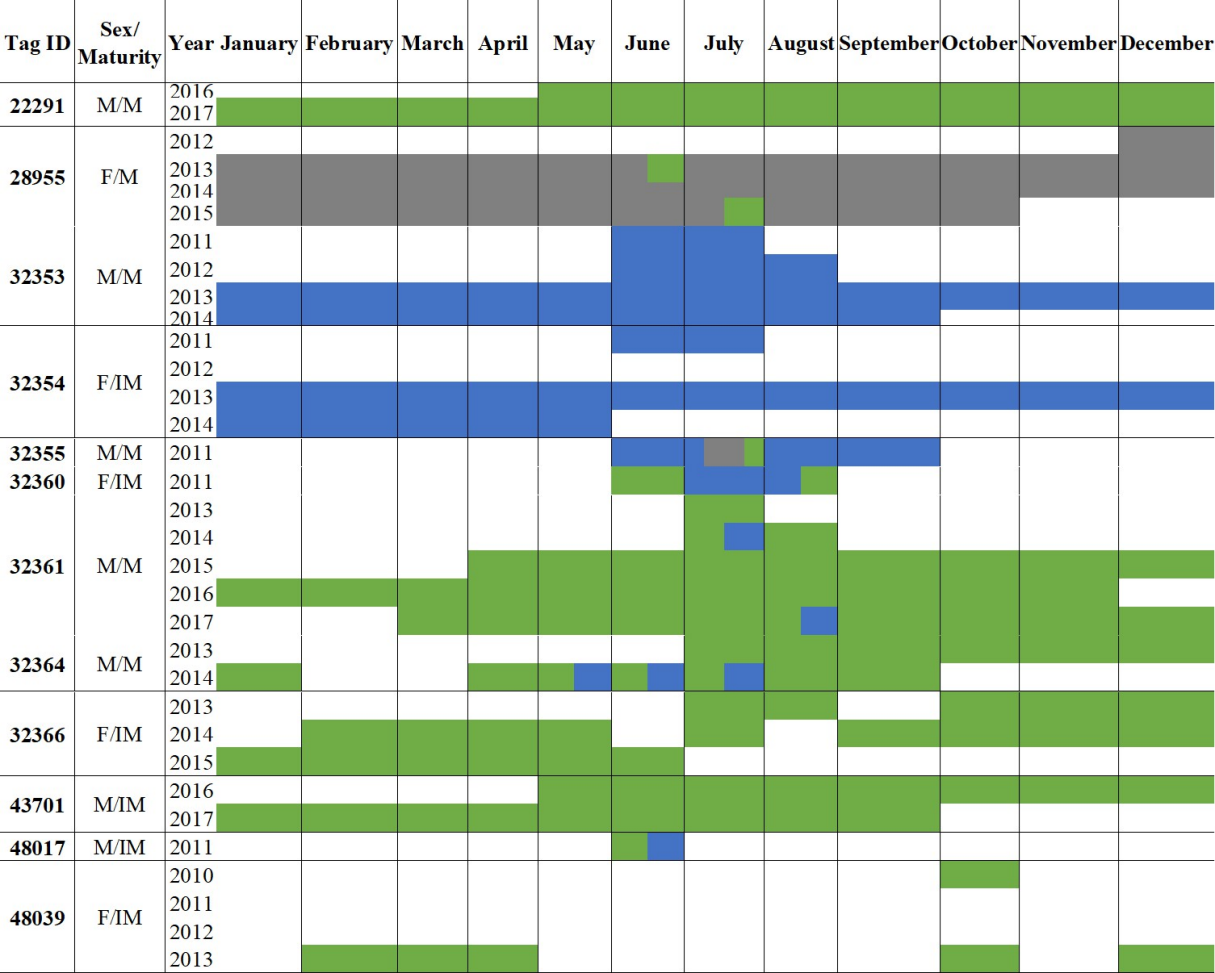
Monthly detection patterns of individual Caribbean reef sharks (sex (F = female, M = male), maturity (IM = immature, M = mature)) with ≥ one year detection period or that visited ≥ two islands (n = 12) during 2010–2019. The colours indicate which islands each individual was detected on at different months. Grey = Grand Cayman, green = Little Cayman and blue = Cayman Brac. Note that where individuals were detected over more than one year this is indicated by more than one line of data.

**Fig 11:**
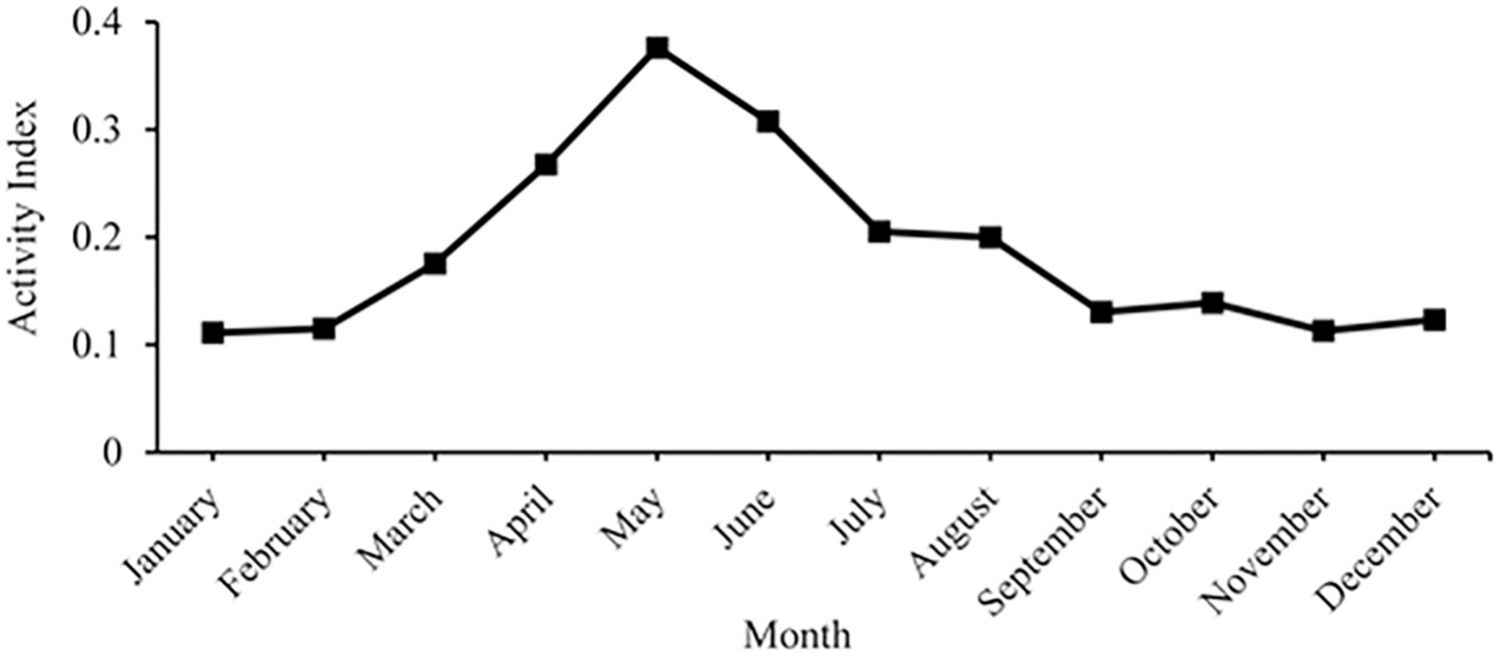
Monthly Activity Index (AI = proportion of detection days times the proportion of detected sharks) of Caribbean reef shark (n = 39) within the entire acoustic array across the whole study period (2010–2019).

Caribbean reef sharks exhibited seasonality in both movement and activity. Coastal space use differed significantly between seasons (Mantle test: r = 0.3477, p < 0.001) and sharks were significantly more mobile (Mann-Whitney U test: W = 1779, p < 0.001) in summer than in winter (summer: 45 receivers, winter: 35 receivers; Fig 9). Upon visual comparison, it appears that all inter-island movements occurred in summer (April–September, Figs 9A and 10) and that shark activity (Activity Index (AI) = proportion of detection days times the proportion of detected sharks) also increased during these months (Figs 9A and 11). Based on monthly detection patterns of individuals (n = 12 sharks) and on values of AI, it appears that the pattern of eight sharks (28955, 32353, 32355, 32360, 32361, 32364, 32366, 48017) showed inter-island movements during May–August, with a peak in July (Fig 10) and an increase in detections during April–September), with a peak in May (Fig 11). The remaining four sharks (22291, 32354, 43701, 48039) remained on the same island and showed no seasonal pattern in detections (Fig 10). It is of note that two sharks, a mature male (32361) and mature female (28955), repeated their inter-island movement in two, non-consecutive summers (Fig 10). The mature male moved from Little Cayman to Cayman Brac in July of one year and, three years later, repeated the trip in August. The mature female moved twice from Grand Cayman to Little Cayman, with the first trip in June and the second in July two years later. During winter (October–March), individuals were generally less active (Fig 11), showing less frequent movement between receivers on the same island. (Fig 9B).

#### Diel movements

There was no clear evidence of diel behaviour in the tagged Caribbean reef sharks. Individuals were detected on the same number of receivers during day and night (n = 37) and both diel periods had a similar total number of detections (day: 44%, night: 56%). There was no significant difference (100% overlap; Mantle test: r = 1, p < 0.001) in coastal space use between diel periods and mobility of sharks did not differ significantly (Mann-Whitney U test: W = 1152, p = 1) between day and night.

## Discussion

Implanted acoustic transmitters (‘tags’) and a non-overlapping acoustic receiver (‘receiver’) array have shown to be a useful method for long-term monitoring of the movement patterns of Caribbean reef sharks in the Cayman Islands; 59% of the sharks tagged were detected, for detection periods of up to 1,598 days (> 4 yrs). The demographic proportions of the sharks tagged were unbiased. The numbers of sharks detected for each sex and maturity stage were limited. Detection of the extent of movement of the sharks, especially at fine scale, will have been conservative due to the coarse spatial coverage and temporal variation of receiver function. The results nevertheless provide key insights into the movement patterns of Caribbean reef sharks in the Cayman Islands, and likely in the Caribbean more generally. This study revealed several clear findings: (1) that individual Caribbean reef sharks were present around the Cayman Islands throughout the year, with no effect of sex or maturity; (2) that while some individuals appeared to be transient visitors, others were resident or pseudo-resident; (3) that the resident individuals showed high site-fidelity to one area of one island; (4) that some individuals made occasional excursions far beyond their home range, with a few moving between all three islands, across open water > 2,000 m depth, showing a minimum displacement of 148.21 km; and (5) that there was an element of seasonal behaviour, with increased movements within and between-island occurring during summer (April–September).

An additional observation was that Caribbean reef sharks in the Cayman Islands appeared to be more frequently detected and more mobile in specific areas of each island. For example, sharks appeared to make more concentrated use of areas N and SE of Grand Cayman, NW, W, and S of Little Cayman, and East and S of Cayman Brac. The concentration of individuals within a certain part of their habitat is prevalent in sharks [76–78] and can be driven by various environmental factors, including food availability [42, 79] and/or anthropogenic activity [39–41, 70, 80]. The more extensive use by the sharks of the south side on each Sister Island may result from their more frequent exposure to wave action and strong currents. Areas with strong current flow are generally ‘hotspots’ for coastal shark species (Caribbean reef shark: [42]; silvertip shark (*Carcharhinus albimarginatus*): [79]; grey reef shark (*Carcharhinus amblyrhynchos*): [81]) since these areas typically offer more productive foraging grounds [82].

At the same time, anthropogenic activity may result in avoidance of some areas by this species, since previous studies have shown that anthropogenic pressure is generally negatively correlated with shark abundance [39–41].On Grand Cayman most human activity, such as boat traffic (e.g. dive boats, fishing charters and cruise ships) as well as recreational water sport activities (e.g. jet skiing, diving), are concentrated along the west and north-west parts of the island, while there is less anthropogenic disturbance along the remainder of the north coast and on the east side. In Brazil, Caribbean reef sharks are less abundant in areas exposed to intense anthropogenic use, including boat traffic and fishing pressure, both of which are thought to cause sharks to avoid these areas [42]. Most likely the distribution of sharks in the Cayman Islands is shaped by a combination of anthropogenic activities (boat traffic, diving pressure), habitat and prey availability.

In investigating levels of residency in this study, we used both the Residency Index (RI) and the Classification of Residency (CR) to avoid bias as a consequence of the limited acoustic coverage. While RI is numerical, allowing for quick and comparable results, it is recognized as a (highly) conservative measure likely to underestimate residency of a species because individuals are likely to move within a study area without being detected by the receiver array [11]. Furthermore, the definition of ‘monitoring period’ varies in the literature and is defined as either the number of days from the date of tagging to the date of last detection [10, 12, 63, 64] or as the number of days expected from the battery life of the tag (‘tag life’, [2, 63, 64, 83]). The difference in definition of the monitoring period by different authors has also recently been highlighted by Appert et al. [63], who conclude that in some species and individuals it may significantly influence the findings. In this study it skews RI values, especially when the numbers of detections are low. For example, the value of RI of a shark that was detected on three days over the first two weeks after tagging and then left the study area (i.e. was not detected again thereafter) is much higher using a monitoring period based on date of last detection (e.g. 14 days) than using maximum tag life (e.g. V16 = 1,915 days). Alternatively, the use of CR based on individual behaviour patterns, adapted from Werry et al. [48], allows one to ‘zoom out’ and ‘smooth-over’ periods of apparent absence, which could be advantageous when detection numbers are low. For example, a shark that was detected on three days only would not be considered ‘resident’ while a shark that was detected sporadically but constantly would be classed as resident. However, CR requires more time for the analysis of overall behaviour patterns and its value is perhaps more limited because it has not yet been used by other researchers since its introduction by Werry et al. [48].

Caribbean reef sharks were captured and detected throughout each year during the nine-year study period, demonstrating that this species has a year-round presence around the coastal shelf edge and is highly resident in Cayman. Surprisingly, the level of residency in this study (RI ≤ 0.43) was much lower than in other reports for reef-associated species including Caribbean reef [2, 11, 28], blacktip (*Carcharhinus limbatus*) [84, 85], blacktip reef (*Carcharhinus melanopterus*) [65], and grey reef shark [49, 79, 86]. This was also reflected in CR results in which only a minority of sharks (19%) were classified as *resident*. Interestingly, the mean RI value recorded was similar to that reported for migratory species such as the great hammerhead shark (*Sphyrna mokarran*) in the US and Bahamas (RI 0.08 ± 0.10 [67]). However, since in this study the monitoring period was calculated using maximum tag life, the mean RI will have been skewed by individuals that died or departed the monitoring area earlier on in the study. That is, these sharks would appear far less resident than using the date of last detection (as used by many studies).

It was also noticeable that in the present study a low percentage of tagged sharks were subsequently detected on the array (59%) compared to those proportions (> 94%) detected in studies in other parts of the Caribbean [2, 11, 23]. It is unlikely that the low residency values were due to fishing or post-tagging mortality, since Caribbean reef sharks are known to be resilient to experimental longline capture [55], and show low levels of post-release mortality [23]. While it is possible that some of the individuals may had lost their tags, for reasons mentioned in the methods section, this seems unlikely. Certainly, it seems implausible that such a high number of tags could have been ejected, and much more likely that these data can be attributed to limited acoustic coverage and/or the departure of individuals from the study area (64%: [87], 30%: [23]). Sporadic absence of highly resident (mean RI = 0.43) Caribbean reef sharks was observed in Belize and it was suggested to be due to occasional departure from the study area [2].

In the Cayman Islands, Caribbean reef sharks (especially mature individuals) tend to swim along the coastal slope, below shelf/reef edge (author, pers. obs. and as reported in previous studies in which instances tagged individuals are not detected by the receivers [20, 27, 57]). The very narrow coastal shelf may result in sharks spending more time at deeper depths close to the coastal shelf, or alternatively, sharks may also spend time inside lagoons (author, pers. obs.) that likewise are not covered by the acoustic array. Such behaviour seems the most plausible explanation for the relatively low detection numbers of some individuals. Considering that in this study the acoustic coverage was modest (7% of the coastal shelf), variable in space and time, and ineffective much below the reef drop-off at 30 m, it seems more likely that the low residency of sharks observed in this study was due to individuals not being detected even though still present in Cayman coastal waters.

While a suggested year-around presence of Caribbean reef sharks in the Cayman Islands was found, this study also demonstrated that individual Caribbean reef sharks exhibit occasional long-distance (> 100 km) movements and across deep (up to 2,000 m) water. These long-distance movements by some sharks between the different Cayman Islands were also evident from parallel work using photo-identification of individuals recorded on BRUVS [22]. Large scale movements (> 100 km) have been observed for other reef-associated species including grey reef shark (250 km [86], 134 km: [53]), blacktip reef shark (138 km: [88]) and bull shark (*Carcharhinus leucas*) (650 km: [49], 2,000 km [89]) and some studies suggested that Caribbean reef sharks might also be more dispersive than previously thought [11, 23]. In this study, some mature sharks (n = 6) moved between Grand Cayman and the Sister Islands and to date this appears to be the first documented movements by Caribbean reef sharks over distances greater than 50 km; the maximum displacement this study recorded was 148 km across open ocean deeper than 2,000 m (see also [22, 24]).

The above observation is important as it suggests potential connectivity of the local population with Caribbean reef sharks within the water of adjacent island nations as is also suggested by genetic studies [21]. The movements recorded here suggest that this species is likely able to travel the distance between the waters not only of the three adjacent island nations of Cayman, but to Cuba and Jamaica (shortest, straight-line distance: Cayman Brac to Jardines de la Reina in Cuba ~ 142 km, Cayman Brac to Jamaica ~ 208 km, Cuba to Jamaica ~ 213 km), despite the deep open ocean between them. Further, the CR data reported here suggest that the majority of Caribbean reef sharks are either migrants (that come and go) or nomads (that pass-by once), spending only limited amount of time in local waters. If Caribbean reef sharks undergo large scale (> 100 km) movements within the Caribbean, individuals may use Cayman as a ‘navigation point’ or ‘pit stop’ en route from one Caribbean region to another.

The results also support previous findings across the geographic range of this species that Caribbean reef sharks exhibited strong site-fidelity [2, 11, 20, 25, 70] to relatively small areas [11, 70]. In this study, Caribbean reef sharks showed high site-fidelity (mean SFI = 86.32%) to their primary receiver site and the mean home range was about 20 km. However, due to the low number of daily detections for some individuals and the detection of sharks on multiple adjacent receivers, individuals may have been detected while travelling or moving between sites, i.e. when displaying roaming rather than home ranging behaviour and site-fidelity. The negative relationship between the SFI value and the distance between each primary receiver and the other receivers that detected the same shark supports the hypothesis of site-fidelity for most Caribbean reef sharks in Cayman. Site-fidelity might reflect benefits associated with a specific habitat due to prey availability, favourable environmental conditions, predator avoidance (e.g. especially in juveniles), territoriality and social interactions, less anthropogenic influence, or some combination of these factors [90, 91].

Although in some cases a receiver was identified as the ‘primary site’ of more than one individual, most were the primary site of only one individual, suggesting perhaps a degree of territorial behaviour or at least of intraspecific competition between individuals for food and space. Intra-specific territoriality in Caribbean reef sharks has been described in the US Virgin Islands (USVI) [28]. A few sharks in that study appeared to exhibit an equally strong affinity to two receivers, some of which were not adjacent suggesting that some individuals show site-fidelity to more than one area. This is similar to movements observed in blacktip reef sharks where individuals moved between ‘patches’ within their core area or home range [92]. Alternatively, site-fidelity may be spatially and temporally dynamic [92], the primary receiver site of an individual may have shifted from one to another receiver site over time. However, data from the present study are not able to give conclusive information on shifting site-fidelity due to the low number of detections and spatiotemporal variation in acoustic coverage.

In the present study, there was also some indication of both ontogenetic expansion of home range size and separation in space use by mature and immature sharks. Minimum linear displacement (MLD) was used to estimate linear home range because the array was not designed for detection ranges to overlap. Comparing home range sizes among studies is difficult, due to differences in temporal scales and metrics used for home range analysis by different authors [90, 93–96], but while Caribbean reef sharks in Belize appeared not to exhibit ontogenetic expansion of home range size [2], such expansion has been observed in juvenile Caribbean reef [11, 70] and other reef sharks elsewhere [65, 79, 93]. While these findings could be an artefact due to the greater detection range of V16 compared to that of V9 tags (used in very small sharks of < 110 cm TL), ontogenetic differences in space use are common in many sharks [9, 10, 28] and thought to be linked to individual energy requirements. Generally, larger individuals have greater energy requirements than smaller ones [97–99] and therefore, need more resources and consequently more space [reviewed in 83, 85, 92]. In contrast, smaller sharks tend to cruise more slowly [98] and to stay within core areas in order to avoid predation [94]; consequently they move between receivers less frequently and avoid movements across deeper water.

In the present study some Caribbean reef sharks (both mature and immature) undertook movements far beyond the usual home range, similar to previous reports of occasional excursions in this species [11, 22, 90, 100]. Such movements by mature individuals could be the result of foraging, social interactions, reproductive requirements, individuals investigating potential foraging grounds [101] while avoiding the territories of other dominant conspecifics [102]. In contrast to the behaviour of mature individuals, immature sharks not only moved strictly between Little Cayman and Cayman Brac, but also seemed to follow a direct path between receivers, that is along the upper reef, through an area with less exposure to potential predators. In fact, immature sharks moving between Little Cayman and Cayman Brac were only detected on a path between the two receivers that were the least distance apart. This observation of some degree of connectivity among immature sharks between the Sister Islands is of interest, given that the islands are separated by a minimum distance of about 5 km and a deep (> 1,000 km) channel. In the present study, two immature females (Fig 10) did not appear on a receiver for one and two years, respectively, but they reappeared on receivers at the same island after their disappearance.

That the pattern of residency of Caribbean reef sharks in Cayman was not influenced by the sex of individuals similar to observations of other reef shark species [79, 81]. However, the weak overlap of space use between females and males suggests a degree of sexual segregation of this species in Cayman. Such sex-based differences in behaviour have been found in several shark species (blacktip reef shark: [65], grey reef shark: [103], whitetip reef shark (*Triaenodon obesus*): [104], and leopard shark (*Triakis semifasciata*): [105]), as well as Caribbean reef sharks [20], and have usually been associated with reproduction or considered to reduce intraspecific competition. In this study, females were detected more frequently on receivers that did not detect males (on Grand Cayman and Cayman Brac) and were detected less frequently on receivers that detected both sexes (on Little Cayman), suggesting that females might seek to avoid male encounters.

Male avoidance by females may mitigate energy demanding mating activities outside of the mating season [106]. In contrast, male sharks were more mobile than females, and generally appeared to exhibit lower levels of site-fidelity; this has been interpreted as a male-biased dispersal strategy similar to mate-searching behaviour in males of other reef sharks [65, 79]. Alternatively, it has been suggested that the higher mobility of males may be a means to reduce intraspecific competition with highly site-attached females [9, 65]. Surprisingly, females in this study, despite having strong site-fidelity, made long-distance movements on more occasions than did males, indicative of possible migrations to more favourable areas for parturition [10, 90] and/or survival of pups.

Our study also provided evidence of seasonal behaviour, with increased shark activity (number of detections and number of sharks) and inter-island movements in summer (March–September), while in winter resident and pseudo-resident individuals only moved between local receivers on each island, presumably within their home range. Increases in abundance during summer are well documented in coastal sharks and are thought to be due increased numbers associated with pupping and reproduction [10, 101, 107], as well as influx of food resource [108] and/or avoidance of unfavourable environmental conditions elsewhere [109, 110]. An increase in abundance in summer was observed in Caribbean reef sharks in The Bahamas [23, 54, 57], but not in juvenile sharks in Brazil [70], most likely due to year-around benefits provided by the nursery area.

Sea surface temperatures (SST) are relatively high (annual mean SST (1985–2019) = 27.8°C), with little temperature change throughout the year (monthly mean SST: 26.2–29.4°C). Most rainfall occurs during the wet season (May to October) with the dry season running from November to April. Tropical storms can occur during the Atlantic hurricane season from June to November, when ocean temperatures are also the highest. Also, since the islands are located at latitude 19–20°N, day length is noticeably longest in late June. These factors would seem to provide clues that could trigger reproductive activity, but unlikely to be sufficient to cause larger scale movement for other reasons.

It is of note that all long-distance movements between islands, especially between Grand Cayman and the Sister Islands, occurred between May and August, peaking in July. Furthermore, only mature sharks moved between Grand Cayman and the Sister Islands; three individuals were detected only during their inter-island movements, and a few sharks did multiple trips between islands. For example, one mature male moved between all three islands in August and another mature male moved from Little Cayman to Cayman Brac and back in three consecutive months (May, June, July). This behaviour coincided with the months when mating scars on female Caribbean reef sharks are evident and mating events, as well as new-born Caribbean reef sharks, (ca. 50 cm) have been observed by divers and on baited remote underwater video systems (BRUVS) [80]. Consequently, it is likely that seasonality is linked to reproductive behaviour of Caribbean reef sharks in Cayman.

This hypothesis is supported by the observation that mature individuals of both sexes undertook repeat trips between islands in non-consecutive years. For example, a mature male moved from Little Cayman to Cayman Brac in July of one year and repeated the trip in August three years later while a mature female moved from Grand Cayman to Little Cayman in July of one year and again two years later. The annual, or in females biennial, reuse of a particular area is a widespread behaviour in large bodied sharks [111] and has been linked to the apparent biennial reproductive cycle of a females in most large-bodied shark species [112], including the Caribbean reef shark, in which the gestation is thought to be approximately one year and females can give birth every two years [113]. Such large-scale movements in mature sharks are thought to assist with finding a mate and suitable habitat for pupping [114, 115].

Interestingly, mature female Caribbean reef sharks moved between Grand Cayman and the Sister Islands on more occasions than males, suggesting potential natal philopatry or at least a preferred mating/pupping area by some females of this species. Natal philopatry, where pregnant females return to their natal region to give birth, has yet to be confirmed in Caribbean reef sharks, however there is evidence that this occurs in lemon sharks (*Negaprion brevirostris*) [116], blacktip shark [117], blacktip reef sharks [101], spottail shark (*Carcharhinus sorrah*) [118] and bull sharks [10, 119]. Nevertheless, in some other shark species, repeated migratory philopatry has been linked to enhanced feeding opportunities [7, 87, 120–123] and thermal conditions [108–110].

Finally, in the present study, sharks did not show any statistically significant patterns of diel occurrence or movement behaviour. This homogenous behaviour is surprising because it was expected that detections, number of individuals detected, and their space use would all increase at night linked to greater foraging activities during the night hours. Although diel behaviour is widespread in sharks and has been linked to various factors including feeding behaviour, predator avoidance [26, 86, 124, 125], energetic advantages [79, 126], and environmental conditions [9, 92], a lack of diel behaviour has also been observed in several other tropical coastal shark species [18, 92, 127]. Elsewhere, Caribbean reef sharks have been reported to exhibit diel shift in habitat with sharks tending to occur deeper (e.g. outer reef, deeper depth) during the day and shallower (e.g. inside lagoons, shallower depth) during the night, behaviour that has been linked to both foraging success and predator avoidance [26, 27].

The present study has provided an understanding of the horizontal movements of Caribbean reef sharks in the Cayman Islands, knowledge of which is important for the conservation and management of the species. MPAs in the Cayman Islands are long established (> 30 yrs) and recently enhanced to cover 48% of the coastal shelf (2021). Although the MPAs have proven to be beneficial for reef fish populations [128], the wide-ranging nature of Caribbean reef sharks revealed by this present study indicates that the home ranges of individual sharks extend to areas outside the protected zones, where they can be exposed to recreational fishing. The realisation that existing MPAs were therefore unlikely to provide adequate protection for threatened shark species was a key factor leading to legislation, introduced in 2015, giving blanket protection to all sharks throughout Cayman waters [32].

## Supporting information

S1 Fig Sketch of a receiver station including concrete anchor, rope (yellow line), sub-surface float and acoustic receiver. Image was created in www.Canva.com.
(TIF)

S2 Fig Sketch of A) scientific longline and B) handline to capture Caribbean reef sharks for acoustic tagging in the Cayman Islands. Image was created in www.Canva.com.
(TIF)

S3 Fig Presence of Caribbean reef sharks (n = 39) in the Cayman Islands during the nine-year study period (2010–2019). Tag IDs are listed in order of deployment date. • indicates tagging month, X indicates ≥ 1 detection day in the month, grey bar represents the estimated tag life in days.
(TIF)

S4 Fig Comparison of mean (± SE) A) detection period in days, B) number of detection days and C) number of detections of Caribbean reef sharks (n = 39) among sex, maturity and capture island. Detection metrics of sharks did not differ significantly between sex (Mann-Whitney U test: detection period: W = 514.5, p = 0.774; detection days: W = 521, p = 0.841; number of detections: W = 515, p = 0.779), maturity (Mann-Whitney U test: detection period: W = 517.5, p = 0.769; detection days: W = 531.5, p = 0.915; number of detections: W = 532, p = 0.920), or capture island (Kruskal-Wallis rank sum test: detection period: χ2 = 1.268, df = 2, p = 0.531; detection days: χ2 = 1.4071, df = 2, p = 0.495; number of detections: χ2 = 1.505, df = 2, p = 0.471).
(TIF)

S5 Fig A) Boxplots of the Site-Fidelity Index (SFI) for primary receivers from female and male Caribbean reef sharks and B) The SFI of primary receivers against the TL of individual sharks, including regression-line and equation. The median values are indicated by the mid-column horizontal bar within the box; X represents mean values; the length of the box is the inter-quartile range; whiskers represent quartiles; white circles are outlier values.
(TIF)

S6 Fig Boxplots of minimum linear displacement (MLD) by contrasting Caribbean reef shark demographics. The median values are indicated by the mid-column horizontal bar within the box; X represents mean values; the length of the box is the inter-quartile range; whiskers represent quartiles; white circles are values.
(TIF)

S1 Table Post-hoc Dunn test results of pairwise comparison of Site-Fidelity Index (SFI) between receiver ranks from tagged Caribbean reef sharks in the Cayman Islands. Test statistic (Z) and p-values are reported and significant differences, at the 0.05 level, are marked with *.
(PDF)
