# Supplementary figures and images for: Individual residency behaviours and seasonal long-distance movements in acoustically tagged Caribbean reef sharks in the Cayman Islands
Source: PLoS One. 2023 Nov 27;18(11):e0293884. doi: 10.1371/journal.pone.0293884 (PMC10681323; doi:10.1371/journal.pone.0293884)

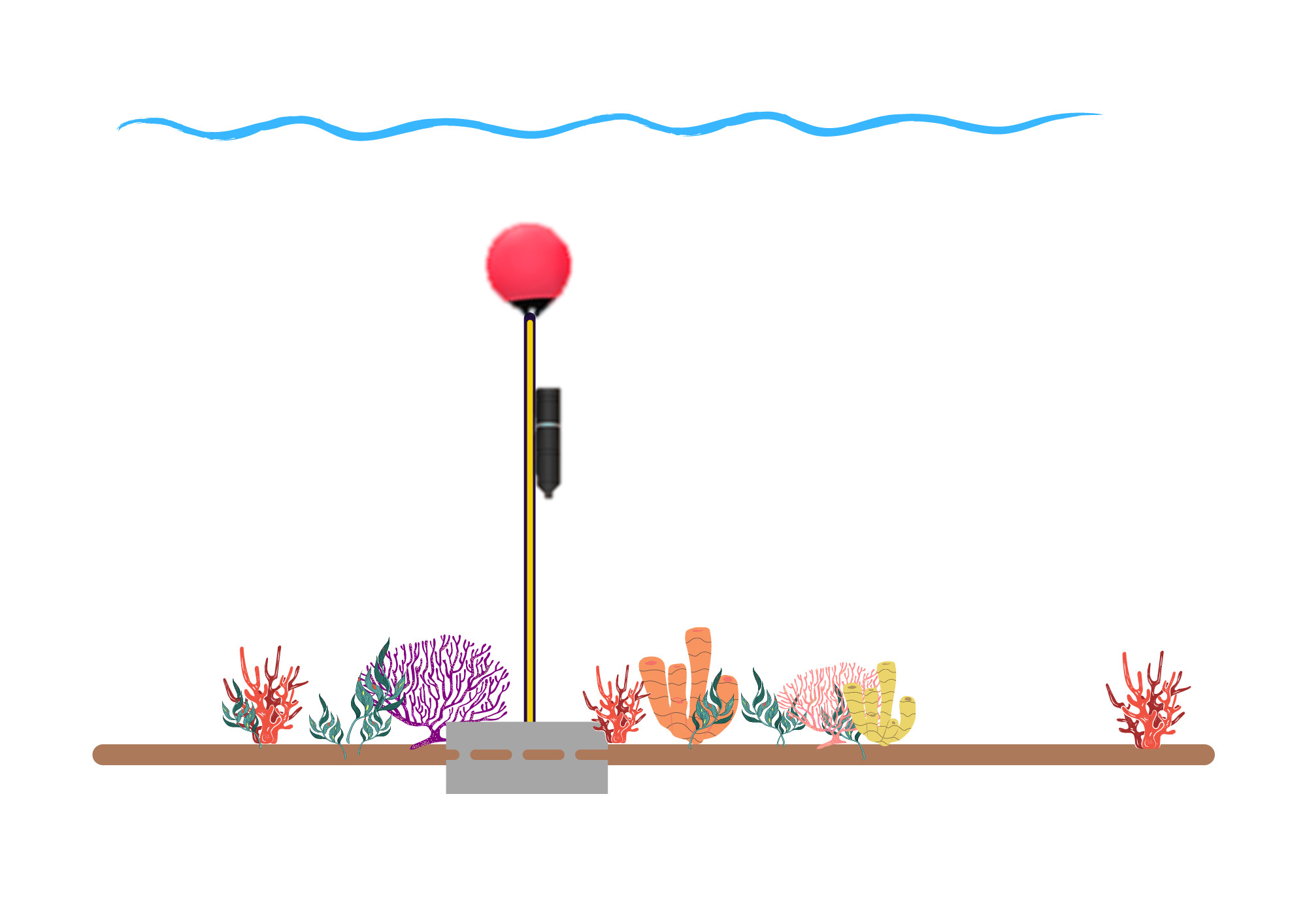

Supplement: S1 Fig — Image was created in www.Canva.com. (TIF) [file pone.0293884.s001.tif]

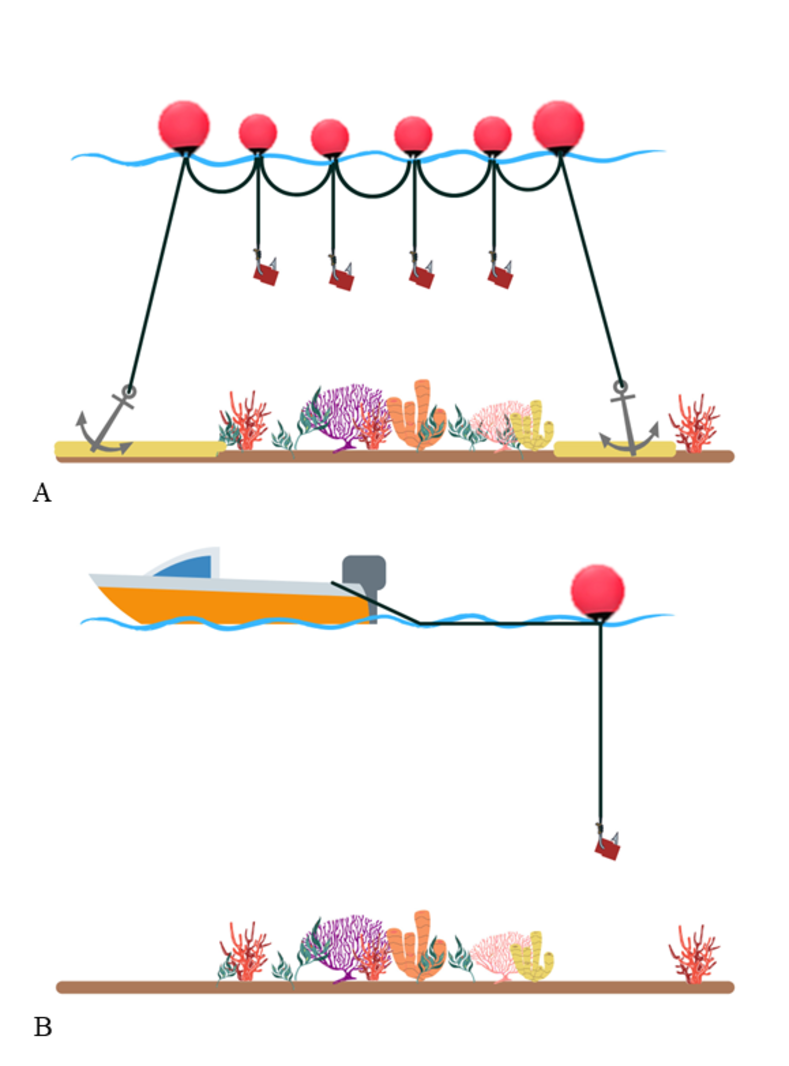

Supplement: S2 Fig — Sketch of A) scientific longline and B) handline to capture Caribbean reef sharks for acoustic tagging in the Cayman Islands. Image was created in www.Canva.com. (TIF) [file pone.0293884.s002.tif]

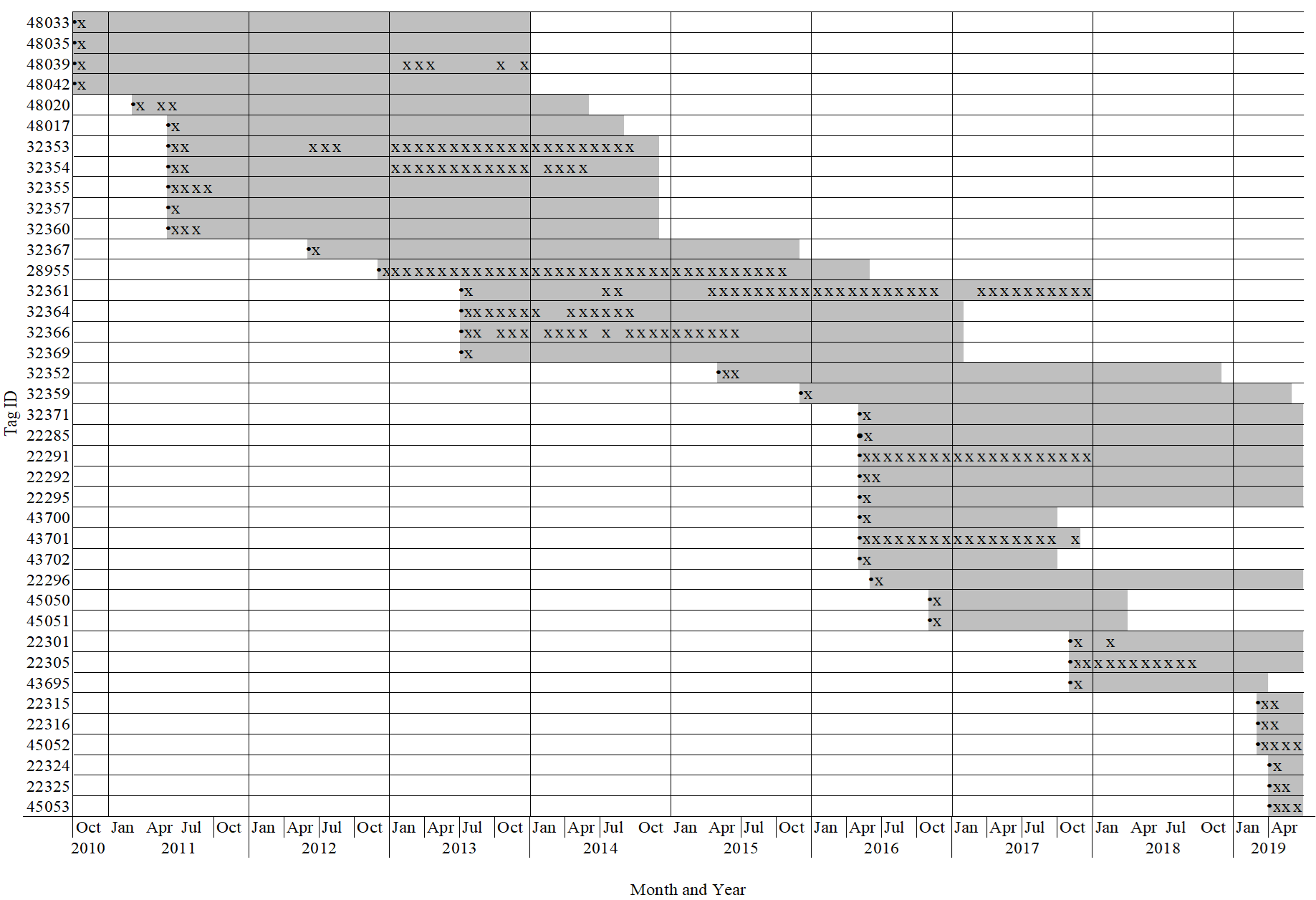

Supplement: S3 Fig — Tag IDs are listed in order of deployment date. • indicates tagging month, X indicates ≥ 1 detection day in the month, grey bar represents the estimated tag life in days. (TIF) [file pone.0293884.s003.tif]

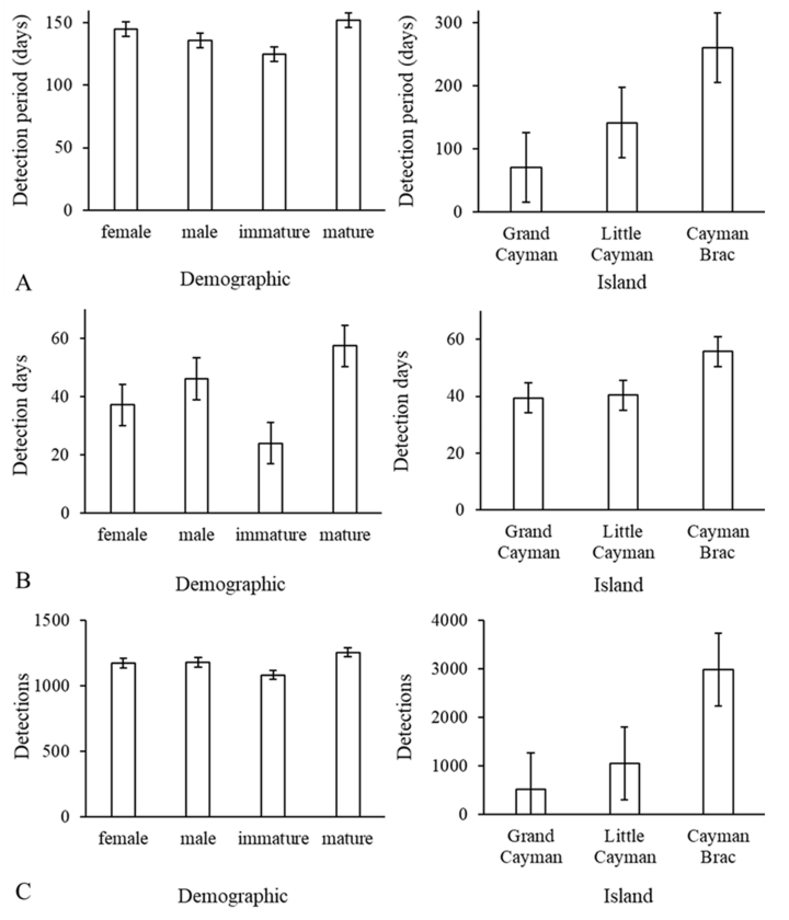

Supplement: S4 Fig — Comparison of mean (± SE) A) detection period in days, B) number of detection days and C) number of detections of Caribbean reef sharks (n = 39) among sex, maturity and capture island. Detection metrics of sharks did not differ significantly between sex (Mann-Whitney U test: detection period: W = 514.5, p = 0.774; detection days: W = 521, p = 0.841; number of detections: W = 515, p = 0.779), maturity (Mann-Whitney U test: detection period: W = 517.5, p = 0.769; detection days: W = 531.5, p = 0.915; number of detections: W = 532, p = 0.920), or capture island (Kruskal-Wallis rank sum test: detection period: χ2 = 1.268, df = 2, p = 0.531; detection days: χ2 = 1.4071, df = 2, p = 0.495; number of detections: χ2 = 1.505, df = 2, p = 0.471). (TIF) [file pone.0293884.s004.tif]

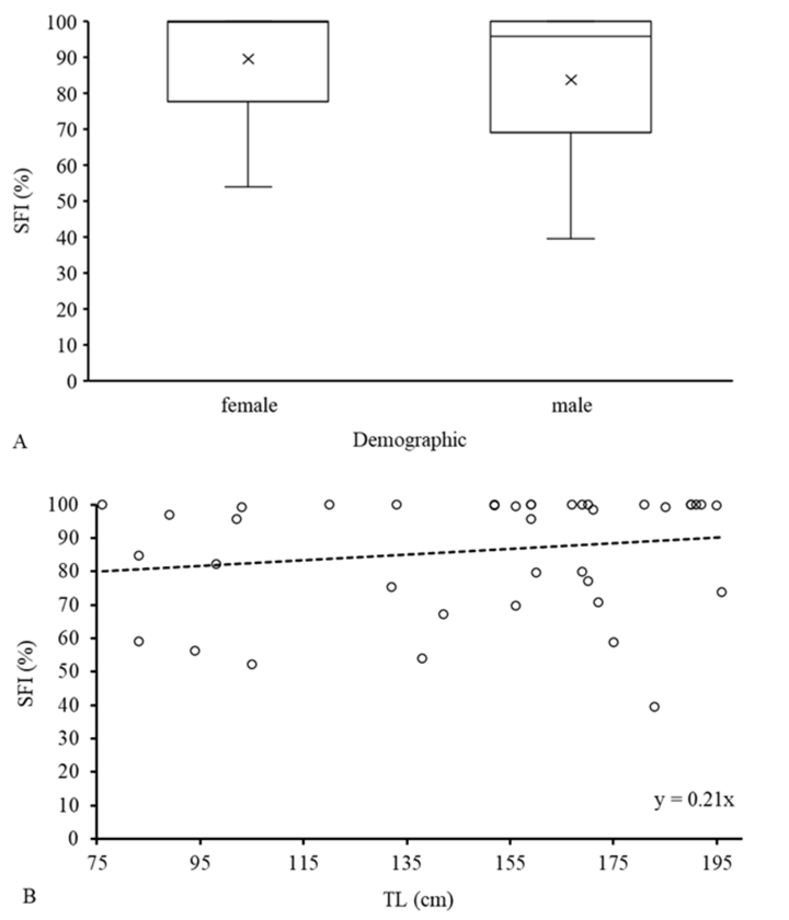

Supplement: S5 Fig — A) Boxplots of the Site-Fidelity Index (SFI) for primary receivers from female and male Caribbean reef sharks and B) The SFI of primary receivers against the TL of individual sharks, including regression-line and equation. The median values are indicated by the mid-column horizontal bar within the box; X represents mean values; the length of the box is the inter-quartile range; whiskers represent quartiles; white circles are outlier values. (TIF) [file pone.0293884.s005.tif]

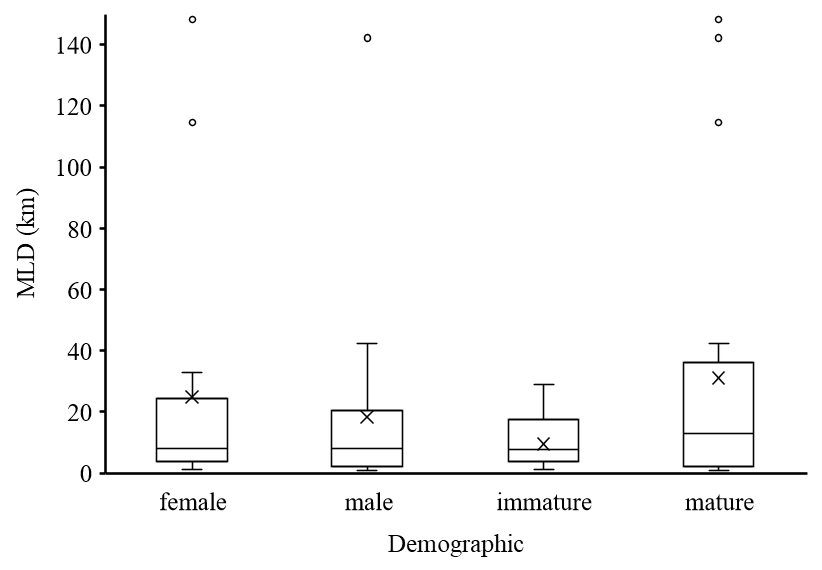

Supplement: S6 Fig — The median values are indicated by the mid-column horizontal bar within the box; X represents mean values; the length of the box is the inter-quartile range; whiskers represent quartiles; white circles are values. (TIF) [file pone.0293884.s006.tif]
