# Supplementary material for: Individual residency behaviours and seasonal long-distance movements in acoustically tagged Caribbean reef sharks in the Cayman Islands
Source: PLoS One. 2023 Nov 27;18(11):e0293884. doi: 10.1371/journal.pone.0293884 (PMC10681323; doi:10.1371/journal.pone.0293884)
Supplement: S1 Table — Test statistic (Z) and p-values are reported and significant differences, at the 0.05 level, are marked with *. (PDF) [file pone.0293884.s007.pdf]

| Receiver rank | Test statistic | 1 | 2 | 3 | 4 | 5 | 6 |
|---|---|---|---|---|---|---|---|
| 2 | Z | 4.060 | | | | | |
| | p | < **0.001*** | | | | | |
| 3 | Z | 4.427 | 0.724 | | | | |
| | p | < **0.001*** | 0.235 | | | | |
| 4 | Z | 4.935 | 1.543 | 0.825 | | | |
| | p | < **0.001*** | 0.061 | 0.205 | | | |
| 5 | Z | 5.051 | 1.917 | 1.231 | 0.433 | | |
| | p | < **0.001*** | **0.028*** | 0.109 | 0.332 | | |
| 6 | Z | 4.441 | 1.934 | 1.378 | 0.707 | 0.327 | |
| | p | < **0.001*** | **0.027*** | 0.084 | 0.240 | 0.372 | |
