# Supplementary material for: Individual residency behaviours and seasonal long-distance movements in acoustically tagged Caribbean reef sharks in the Cayman Islands
Source: PLoS One. 2023 Nov 27;18(11):e0293884. doi: 10.1371/journal.pone.0293884 (PMC10681323; doi:10.1371/journal.pone.0293884)
Supplement: Attachment — Submitted filename: Response to Reviewers_final.docx [file pone.0293884.s008.docx]

*PLOS ONE*

Dear Editor and Reviewers,

Please see my response to each point raised by the academic editor and reviewer(s) below.

1. The manuscript was amended to meet PLOS ONE's style requirements, including those for file naming. Amongst other things, the title, author list and affiliations were removed from MS and put into a separate file and labelled as per guidelines. The following of the manuscript has been corrected:

- Page numbers were amended in order to align bottom left.
- Some of the figure citations in the text were corrected to meet formatting guidelines.
- Some of the figure captions were corrected to meet formatting guidelines.
- The format of each equation was corrected.
- Double spacing was added to the entire text.
- The level of some (sub-)headings was corrected.
- Funding information was removed from the acknowledgements.

2. /
3. The information for ‘Funding Information’ and ‘Financial Disclosure’ sections was corrected, including correct grant numbers and full name of the funders. Please see text below. Please note that the funding information was entered into the online submission system, as instructed during the submission process. However, after carefully checking the final PDF, the wording in the online system does not match paragraph below. I would like to let you know that effort was made to edit the online entry in order to match the wording below, however this was not possible.

This work was supported by the following grants: Save Our Seas Foundation P-145 (2012-2014) (MG) https://saveourseas.com/; Overseas Territories Environment Programme, OTEP-601, OTEP-701 (MG) http://www.ukotcf.org/otep/; United Kingdom Darwin Initiative DPLUS036 (MG) https://www.darwininitiative.org.uk/ and funding from Cayman Islands Brewery Ltd. 2009-2019 (DoE) https://www.cib.ky/caybrew generated through their conservation beer initiative. A small proportion of the income from the sale of the local brewery’s ‘Whitetip Lager’ is donated to the Cayman Islands Department of Environment’s shark research and provided additional funding for this study. The funders had no role in study design, data collection and analysis, decision to publish, or preparation of the manuscript.

4. This reference in MS was corrected to refer to Department of Environment’s (DoE’s) website. After careful reviewing, other references in the text were also corrected.
5. After additional communication with the PLOS ONE editorial office, clarification was provided stating that unique images created in Canva, maps generated in R studio are acceptable to be published under the CC BY 4.0 license without needing written consent.

Relevant figure captions were amended to clarify origin and provide proper attribution where needed.

Relevant figures (networks) and maps were updated to be based solely on DoE data and created by the DoE. Written consent is provided by the DoE Director Gina Ebanks-Petrie.

6. After additional communication with the PLOS ONE editorial office, clarification was provided stating that unique images created in Canva, maps generated in R studio are acceptable to be published under the CC BY 4.0 license without needing written consent.

Relevant figure captions were amended to clarify origin and provide proper attribution where needed.

7. The reference list was checked and amended where necessary.
8. Reply to the additional editor comment:

Link errors on pages 22 and 23 were corrected. The resubmitted PDF was checked that these figures are shown correctly in the text.

Sincerely,

Dr. Johanna Kohler

Cayman Islands Department of Environment

[Johanna.k.kohler@gmail.com](mailto:Johanna.k.kohler@gmail.com), +1 (345) 926-0135
