# Supplementary material for: Individual residency behaviours and seasonal long-distance movements in acoustically tagged Caribbean reef sharks in the Cayman Islands
Source: PLoS One. 2023 Nov 27;18(11):e0293884. doi: 10.1371/journal.pone.0293884 (PMC10681323; doi:10.1371/journal.pone.0293884)
Supplement: Attachment — Submitted filename: PLOS ONE edits_response to reviewers_final.docx [file pone.0293884.s009.docx]

PLOS ONE

Dear Editor and Reviewers,

Please find the responses to each point raised by the reviewers below.

**Reviewer #1**

The numbering of figures was reviewed and corrected where needed.

The references to figures in the text were corrected. The figures were cross references in the word document, but it seems this format causes trouble when converted into PDF, unfortunately. Now the numbering was manually entered which hopefully results in correct numbering in the PDF as well. Please note, figures with “S and number” are referring to figures in the Supporting Info.

The references regarding ‘Vemco Ltd’ were amended to ‘Innovasea Systems Inc.’.

The Ethics statement was amended to match the information in the method section.

We do not have any evidence of tag shedding. Dicken et al.2006 wrote about tag shedding in external tags with an insert point, but we are not aware of any studies suggesting that tags surgically inserted under the skin are shed. Unlike e.g. cat skin, shark skin layers are adhesive allowing the tag to remain in place. It would be pretty difficult for the rounded tag to emerge, the surface of the tag also being of a substance to reduce any reaction by the shark. If the tag had been inserted into the coelum, as some researchers do, then there is a good chance of the tag not only moving, but possibly causing an obstruction. Nevertheless, this is a good point, and it has been addressed in the Method section (starting line 205) and Discussion (starting line 703) to make it clearer.

*Dicken, M. L., Booth, A. J., and Smale, M. J. (2006). Preliminary observations of tag shedding, tag reporting, tag wounds, and tag biofouling for raggedtooth sharks (Carcharias taurus) tagged off the east coast of South Africa. ICES J. Mar. Sci. 63, 1640–1648. doi: 10.1016/j.icesjms.2006.06.009.*

**Reviewer #2**

Line 44/46: The sentence was amended to make it clearer (now line 42-45).

Line 71: The sentence was amended to make it clearer.

Line 77: The sentence was amended as suggested (now line 79).

Line 80: “linear” was added before “home range”.

Line 85: Sentence was amended as suggested (now line 87).

Line 125: Figure caption was amended to include number of receivers (now line 119).

Fig2: Was moved to supplementary material.

Line 138-142 and 151-156 (range tests): These are good points. The two paragraphs were amended to make this clearer.

Fig3: Was moved to supplementary material.

Lines 259-261: Relevant section was removed. We did not get any duplicates on the same receiver. It was part of the protocol, but perhaps because the array was not overlapping there was no need for this step. (now line 265)

Line 285: “s” was deleted. (now line 288)

Lines 292-298: Appert et al., 2023 and Kraft et al., 2023 were added to the references.

Lines 299- 311: The differences that Appert et al., 2023 and Kraft et al 2023 address are briefly mentioned in the Discussion. So instead of discussing the issue of different equations and the consequences for the calculation of RI values here, the relevant section in the Discussion was amended to expand on this topic and to cite both studies (starting line 555). Here, Kraft et al. and Appert et al. were added to the in-text citation (line 304-306).

Line 346: This point is valid and we did consider redoing part of the data analyses, in particular the values for MLD, accounting for a shorter transmission range of the V9 tags. However, after consultation with Innovasea, we decided not to make any changes to the data analyses. While the range would certainly be less for a V9 compared to a V16 tag, the relationship is not linear. That means that *theoretically* an increase of 3dB is double the power but that this does not equate to twice the distance. Hence it is not possible to estimate the V9 range given what we had found for a V16 tag. That being said, we did test for differences between mature and juvenile sharks (most of which were fitted with V9s) and did not find any significant differences - this is mentioned in the MS. We do appreciate the concern raised by the reviewer and to make it clearer, the MS was amended mentioning the difference in transmission range between the V16 and V9 tags in the Methods, Results and Discussion.

Line 355: The sentence was amended to make it clearer.

Line 464/465: The last sentence in the paragraph mentioned that there was no difference between maturity, assuming most of these individuals were fitted with V9 tags. Although this is an interesting and valid point, no changes were made because of the reasons mentioned above.

Fig S1: No changes were made. The overall feedback by reviewers was that MS is ‘figure heavy’. While we believe that the figures are beneficial for the reader to understand and visualize the results, we appreciate the option for readers to view these from the Supporting Information.

Line 560-562: A sentence was added to mention this point. Furthermore, a sentence was added to the Discussion to make it clearer (see line 777).

Would it be possible that “passer-by” sharks were captured or died?: This possibility is mentioned in the Discussion (see line 699). This paragraph was amended to make it clearer.

Lines 682-690: The suggested references were added.

Line 758: While this paper uses linear home range, “home range” was not mentioned here. No changes were made.

Line 826-834: A paragraph was added to provide information on Cayman’s summer/winter conditions (starting line 826).

**Reviewer #3**

Abstract

Line 44-46: A brief description on the types of analyses used to assess the various aspects of space use and site fidelity was added to the Abstract.

Tables/Figures: While we believe that the figures are beneficial for the reader to understand and visualize the results, we appreciate the option for readers to view these from the Supporting Information. In response to your specific suggestions, the following changes were made:

-Figures 9, 10, and 11 were kept separate. We believe it would be difficult to see details if all networks would be combined.

-Figure 3: Was moved to Supporting info.

-References to Figures 1, 2, and 3 and overall numbering was corrected. The figures were cross references in the word document, but it seems this format caused trouble when converting into PDF, unfortunately. Now the numbering was manually entered which hopefully results in correct numbering in the PDF as well. Please note, figures with “S and number” are referring to figures in the Supporting Info.

**PLOS ONE**

PLOS Figures: Figures were amended via PACE and the corrected versions were uploaded.

Sincerely,

Dr. Johanna Kohler

Cayman Islands Department of the Environment

[Johanna.k.kohler@gmail.com](mailto:Johanna.k.kohler@gmail.com), +1 (345) 926-0135
