# Supplementary material for: Individual residency behaviours and seasonal long-distance movements in acoustically tagged Caribbean reef sharks in the Cayman Islands
Source: PLoS One. 2023 Nov 27;18(11):e0293884. doi: 10.1371/journal.pone.0293884 (PMC10681323; doi:10.1371/journal.pone.0293884)
Supplement: Peer review history

# Editor report: Decision Letter 0

**DOI:** 10.1371/journal.pone.0293884.r001

**Authors:** Rui Coelho

31 May 2023

PONE-D-23-13313Individual residency behaviors and seasonal long-distance movements in acoustically tagged Caribbean reef sharks in the Cayman IslandsPLOS ONE

Dear Dr. Kohler,

Thank you for submitting your manuscript to PLOS ONE. After careful consideration, we feel that it has merit but does not fully meet PLOS ONE’s publication criteria as it currently stands. Therefore, we invite you to submit a revised version of the manuscript that addresses the points raised during the review process.

Please submit your revised manuscript by Jul 15 2023 11:59PM. If you will need more time than this to complete your revisions, please reply to this message or contact the journal office at plosone@plos.org. When you're ready to submit your revision, log on to https://www.editorialmanager.com/pone/ and select the 'Submissions Needing Revision' folder to locate your manuscript file.

Please include the following items when submitting your revised manuscript:

- A rebuttal letter that responds to each point raised by the academic editor and reviewer(s). You should upload this letter as a separate file labeled 'Response to Reviewers'.
- A marked-up copy of your manuscript that highlights changes made to the original version. You should upload this as a separate file labeled 'Revised Manuscript with Track Changes'.
- An unmarked version of your revised paper without tracked changes. You should upload this as a separate file labeled 'Manuscript'.

If you would like to make changes to your financial disclosure, please include your updated statement in your cover letter. Guidelines for resubmitting your figure files are available below the reviewer comments at the end of this letter.

If applicable, we recommend that you deposit your laboratory protocols in protocols.io to enhance the reproducibility of your results. Protocols.io assigns your protocol its own identifier (DOI) so that it can be cited independently in the future. For instructions see: https://journals.plos.org/plosone/s/submission-guidelines#loc-laboratory-protocols. Additionally, PLOS ONE offers an option for publishing peer-reviewed Lab Protocol articles, which describe protocols hosted on protocols.io. Read more information on sharing protocols at https://plos.org/protocols?utm_medium=editorial-email&utm_source=authorletters&utm_campaign=protocols.

We look forward to receiving your revised manuscript.

Kind regards,

Rui Coelho, PhD

Academic Editor

PLOS ONE

Journal Requirements:

When submitting your revision, we need you to address these additional requirements.

1. Please ensure that your manuscript meets PLOS ONE's style requirements, including those for file naming. The PLOS ONE style templates can be found at 

https://journals.plos.org/plosone/s/file?id=wjVg/PLOSOne_formatting_sample_main_body.pdf and 

https://journals.plos.org/plosone/s/file?id=ba62/PLOSOne_formatting_sample_title_authors_affiliations.pdf

2. We note that the grant information you provided in the ‘Funding Information’ and ‘Financial Disclosure’ sections do not match. 

When you resubmit, please ensure that you provide the correct grant numbers for the awards you received for your study in the ‘Funding Information’ section.

3. Please expand the acronym “SOSF” (as indicated in your financial disclosure) so that it states the name of your funders in full.

This information should be included in your cover letter; we will change the online submission form on your behalf.

4. We note that you have referenced (DoE, unpublished data) on page 5 which has currently not yet been accepted for publication. Please respond by return e-mail with a copy of your updated manuscript to include to remove this from your References and amend this to state in the body of your manuscript: (ie “Bewick et al. [Unpublished]”) as detailed online in our guide for authors

http://journals.plos.org/plosone/s/submission-guidelines#loc-reference-style.   We can then upload this to your submission on your behalf.

5. We note that Figures 1, 7, 9, 10, 11 and 14 in your submission contain [map/satellite] images which may be copyrighted. All PLOS content is published under the Creative Commons Attribution License (CC BY 4.0), which means that the manuscript, images, and Supporting Information files will be freely available online, and any third party is permitted to access, download, copy, distribute, and use these materials in any way, even commercially, with proper attribution. For these reasons, we cannot publish previously copyrighted maps or satellite images created using proprietary data, such as Google software (Google Maps, Street View, and Earth). For more information, see our copyright guidelines: http://journals.plos.org/plosone/s/licenses-and-copyright.

We require you to either (1) present written permission from the copyright holder to publish these figures specifically under the CC BY 4.0 license, or (2) remove the figures from your submission:

a. You may seek permission from the original copyright holder of Figures 1, 7, 9, 10, 11 and 14 to publish the content specifically under the CC BY 4.0 license.  

We recommend that you contact the original copyright holder with the Content Permission Form (http://journals.plos.org/plosone/s/file?id=7c09/content-permission-form.pdf) and the following text:

“I request permission for the open-access journal PLOS ONE to publish XXX under the Creative Commons Attribution License (CCAL) CC BY 4.0 (http://creativecommons.org/licenses/by/4.0/). Please be aware that this license allows unrestricted use and distribution, even commercially, by third parties. Please reply and provide explicit written permission to publish XXX under a CC BY license and complete the attached form.”

Please upload the completed Content Permission Form or other proof of granted permissions as an "Other" file with your submission.

In the figure caption of the copyrighted figure, please include the following text: “Reprinted from [ref] under a CC BY license, with permission from [name of publisher], original copyright [original copyright year].”

b. If you are unable to obtain permission from the original copyright holder to publish these figures under the CC BY 4.0 license or if the copyright holder’s requirements are incompatible with the CC BY 4.0 license, please either i) remove the figure or ii) supply a replacement figure that complies with the CC BY 4.0 license. Please check copyright information on all replacement figures and update the figure caption with source information. If applicable, please specify in the figure caption text when a figure is similar but not identical to the original image and is therefore for illustrative purposes only.

The following resources for replacing copyrighted map figures may be helpful:

USGS National Map Viewer (public domain): http://viewer.nationalmap.gov/viewer/

The Gateway to Astronaut Photography of Earth (public domain): http://eol.jsc.nasa.gov/sseop/clickmap/

Maps at the CIA (public domain): https://www.cia.gov/library/publications/the-world-factbook/index.html and https://www.cia.gov/library/publications/cia-maps-publications/index.html

NASA Earth Observatory (public domain): http://earthobservatory.nasa.gov/

Landsat: http://landsat.visibleearth.nasa.gov/

USGS EROS (Earth Resources Observatory and Science (EROS) Center) (public domain): http://eros.usgs.gov/#

Natural Earth (public domain): http://www.naturalearthdata.com/

6. We note that Figures 2, 3a and 3b in your submission contain copyrighted images. All PLOS content is published under the Creative Commons Attribution License (CC BY 4.0), which means that the manuscript, images, and Supporting Information files will be freely available online, and any third party is permitted to access, download, copy, distribute, and use these materials in any way, even commercially, with proper attribution. For more information, see our copyright guidelines: http://journals.plos.org/plosone/s/licenses-and-copyright.

We require you to either (1) present written permission from the copyright holder to publish these figures specifically under the CC BY 4.0 license, or (2) remove the figures from your submission:

a. You may seek permission from the original copyright holder of Figures 2, 3a and 3b to publish the content specifically under the CC BY 4.0 license. 

We recommend that you contact the original copyright holder with the Content Permission Form (http://journals.plos.org/plosone/s/file?id=7c09/content-permission-form.pdf) and the following text:

“I request permission for the open-access journal PLOS ONE to publish XXX under the Creative Commons Attribution License (CCAL) CC BY 4.0 (http://creativecommons.org/licenses/by/4.0/). Please be aware that this license allows unrestricted use and distribution, even commercially, by third parties. Please reply and provide explicit written permission to publish XXX under a CC BY license and complete the attached form.”

Please upload the completed Content Permission Form or other proof of granted permissions as an "Other" file with your submission. 

In the figure caption of the copyrighted figure, please include the following text: “Reprinted from [ref] under a CC BY license, with permission from [name of publisher], original copyright [original copyright year].”

b. If you are unable to obtain permission from the original copyright holder to publish these figures under the CC BY 4.0 license or if the copyright holder’s requirements are incompatible with the CC BY 4.0 license, please either i) remove the figure or ii) supply a replacement figure that complies with the CC BY 4.0 license. Please check copyright information on all replacement figures and update the figure caption with source information. If applicable, please specify in the figure caption text when a figure is similar but not identical to the original image and is therefore for illustrative purposes only.

7. Please review your reference list to ensure that it is complete and correct. If you have cited papers that have been retracted, please include the rationale for doing so in the manuscript text, or remove these references and replace them with relevant current references. Any changes to the reference list should be mentioned in the rebuttal letter that accompanies your revised manuscript. If you need to cite a retracted article, indicate the article’s retracted status in the References list and also include a citation and full reference for the retraction notice.

Additional Editor Comments:

Dear authors.

I have received and I am handling the paper “Individual residency behaviors and seasonal long-distance movements in acoustically tagged Caribbean reef sharks in the Cayman Islands” with the manuscript reference PONE-D-23-13313, that you submitted for consideration in Plos One.

After an initial read, I believe that the paper is of quality to proceed to the peer-review process. However, I noted that on pages 22 and 23 of the PDF, there are several link errors, that appear in those pages as "Error! Reference source not found." I suppose this may have been some type of automatic link in word or similar, that failed when then PDF was compiled and was not detected by you before final PDF approval and submission.

Those link errors do make the revision a little more difficult, as usually the reviewers use that final compiled PDF for their revisions. I would therefore prefer that you first revise this and compile a new corrected PDF, that can be sent for revision without those missing links.

As such, and afer consultation with the Plos One Editorial office on how to proceed, I am sending the paper back with “minor revisions” and request that you address this issue, and provide a new PDF without such errors. Note that the paper has not yet been peer-reviewed, so this is just a minor editorial revision request, before sending the paper for peer-revision.

Thank you, and please let me know if you have any questions.

Best regards,

Rui Coelho

[Note: HTML markup is below. Please do not edit.]

[NOTE: If reviewer comments were submitted as an attachment file, they will be attached to this email and accessible via the submission site. Please log into your account, locate the manuscript record, and check for the action link "View Attachments". If this link does not appear, there are no attachment files.]

While revising your submission, please upload your figure files to the Preflight Analysis and Conversion Engine (PACE) digital diagnostic tool, https://pacev2.apexcovantage.com/. PACE helps ensure that figures meet PLOS requirements. To use PACE, you must first register as a user. Registration is free. Then, login and navigate to the UPLOAD tab, where you will find detailed instructions on how to use the tool. If you encounter any issues or have any questions when using PACE, please email PLOS at figures@plos.org. Please note that Supporting Information files do not need this step.

# Author comment: Author response to Decision Letter 0

**DOI:** 10.1371/journal.pone.0293884.r002

25 Jul 2023

Dear Editor and Reviewers,

Please see my response to each point raised by the academic editor and reviewer(s) below.

1. The manuscript was amended to meet PLOS ONE's style requirements, including those for file naming. Amongst other things, the title, author list and affiliations were removed from MS and put into a separate file and labelled as per guidelines. The following of the manuscript has been corrected:

- Page numbers were amended in order to align bottom left.

- Some of the figure citations in the text were corrected to meet formatting guidelines.

- Some of the figure captions were corrected to meet formatting guidelines.

- The format of each equation was corrected.

- Double spacing was added to the entire text.

- The level of some (sub-)headings was corrected.

- Funding information was removed from the acknowledgements.

2. /

3. The information for ‘Funding Information’ and ‘Financial Disclosure’ sections was corrected, including correct grant numbers and full name of the funders. Please see text below. Please note that the funding information was entered into the online submission system, as instructed during the submission process. However, after carefully checking the final PDF, the wording in the online system does not match paragraph below. I would like to let you know that effort was made to edit the online entry in order to match the wording below, however this was not possible.

This work was supported by the following grants: Save Our Seas Foundation P-145 (2012-2014) (MG) https://saveourseas.com/; Overseas Territories Environment Programme, OTEP-601, OTEP-701 (MG) http://www.ukotcf.org/otep/; United Kingdom Darwin Initiative DPLUS036 (MG) https://www.darwininitiative.org.uk/ and funding from Cayman Islands Brewery Ltd. 2009-2019 (DoE) https://www.cib.ky/caybrew generated through their conservation beer initiative. A small proportion of the income from the sale of the local brewery’s ‘Whitetip Lager’ is donated to the Cayman Islands Department of Environment’s shark research and provided additional funding for this study. The funders had no role in study design, data collection and analysis, decision to publish, or preparation of the manuscript.

4. This reference in MS was corrected to refer to Department of Environment’s (DoE’s) website. After careful reviewing, other references in the text were also corrected.

5. After additional communication with the PLOS ONE editorial office, clarification was provided stating that unique images created in Canva, maps generated in R studio are acceptable to be published under the CC BY 4.0 license without needing written consent.

Relevant figure captions were amended to clarify origin and provide proper attribution where needed. 

Relevant figures (networks) and maps were updated to be based solely on DoE data and created by the DoE. Written consent is provided by the DoE Director Gina Ebanks-Petrie.

6. After additional communication with the PLOS ONE editorial office, clarification was provided stating that unique images created in Canva, maps generated in R studio are acceptable to be published under the CC BY 4.0 license without needing written consent.

Relevant figure captions were amended to clarify origin and provide proper attribution where needed.

7. The reference list was checked and amended where necessary.

8. Reply to the additional editor comment:

Link errors on pages 22 and 23 were corrected. The resubmitted PDF was checked that these figures are shown correctly in the text.

Sincerely,

Dr. Johanna Kohler

Cayman Islands Department of Environment

Johanna.k.kohler@gmail.com, +1 (345) 926-0135

Attachment Submitted filename: Response to Reviewers_final.docx Click here for additional data file.

# Peer review: Decision Letter 1

**DOI:** 10.1371/journal.pone.0293884.r003

**Authors:** Rui Coelho

28 Sep 2023

PONE-D-23-13313R1Individual residency behaviours and seasonal long-distance movements in acoustically tagged Caribbean reef sharks in the Cayman IslandsPLOS ONE

Dear Dr. Kohler,

Thank you for submitting your manuscript to PLOS ONE. After careful consideration, we feel that it has merit but does not fully meet PLOS ONE’s publication criteria as it currently stands. Therefore, we invite you to submit a revised version of the manuscript that addresses the points raised during the review process. The paper has now been reviewed by 3 referees, and all are supportive of publication. Two of the referees are proposing minor changes, while one referee could not complete the revision as there seems to be still some issues with format error between figures sequence order and text.

At this stage, I am suggesting major changes. I ask you to please address the revisions that have been provided in this initial round by the 3 referees, and also that in the revised version to be submitted, please assure that any remaining issues with figure numbering and sequence are resolved. Once that is accomplished, I will send the paper for a further round of revisions. Please let me know if you have any questions.

Please submit your revised manuscript by Nov 12 2023 11:59PM. If you will need more time than this to complete your revisions, please reply to this message or contact the journal office at plosone@plos.org. When you're ready to submit your revision, log on to https://www.editorialmanager.com/pone/ and select the 'Submissions Needing Revision' folder to locate your manuscript file.

Please include the following items when submitting your revised manuscript:

- A rebuttal letter that responds to each point raised by the academic editor and reviewer(s). You should upload this letter as a separate file labeled 'Response to Reviewers'.
- A marked-up copy of your manuscript that highlights changes made to the original version. You should upload this as a separate file labeled 'Revised Manuscript with Track Changes'.
- An unmarked version of your revised paper without tracked changes. You should upload this as a separate file labeled 'Manuscript'.

If you would like to make changes to your financial disclosure, please include your updated statement in your cover letter. Guidelines for resubmitting your figure files are available below the reviewer comments at the end of this letter.

If applicable, we recommend that you deposit your laboratory protocols in protocols.io to enhance the reproducibility of your results. Protocols.io assigns your protocol its own identifier (DOI) so that it can be cited independently in the future. For instructions see: https://journals.plos.org/plosone/s/submission-guidelines#loc-laboratory-protocols. Additionally, PLOS ONE offers an option for publishing peer-reviewed Lab Protocol articles, which describe protocols hosted on protocols.io. Read more information on sharing protocols at https://plos.org/protocols?utm_medium=editorial-email&utm_source=authorletters&utm_campaign=protocols.

We look forward to receiving your revised manuscript.

Kind regards,

Rui Coelho, PhD

Academic Editor

PLOS ONE

Additional Editor Comments :

Dear Dr. Kohler,

Thank you for the submission of paper “Individual residency behaviours and seasonal long-distance movements in acoustically tagged Caribbean reef sharks in the Cayman Islands” for consideration to publication in PlosOne.

The paper has now been reviewed by 3 referees, and all are supportive of publication. Two of the referees are proposing minor changes, while one referee could not complete the revision as there seems to be still some issues with format error between figures sequence order and text.

At this stage, I am suggesting major changes. I ask you to please address the revisions that have been provided in this initial round by the 3 referees, and also that in the revised version to be submitted, please assure that any remaining issues with figure numbering and sequence are resolved. Once that is accomplished, I will send the paper for a further round of revisions.

Please let me know if you have any questions.

Best regards,

Rui Coelho

[Note: HTML markup is below. Please do not edit.]

Reviewers' comments:

Reviewer's Responses to Questions

**Comments to the Author**

1. If the authors have adequately addressed your comments raised in a previous round of review and you feel that this manuscript is now acceptable for publication, you may indicate that here to bypass the “Comments to the Author” section, enter your conflict of interest statement in the “Confidential to Editor” section, and submit your "Accept" recommendation.

Reviewer #1: (No Response)

Reviewer #2: All comments have been addressed

Reviewer #3: (No Response)

**********

2. Is the manuscript technically sound, and do the data support the conclusions?

The manuscript must describe a technically sound piece of scientific research with data that supports the conclusions. Experiments must have been conducted rigorously, with appropriate controls, replication, and sample sizes. The conclusions must be drawn appropriately based on the data presented. 

Reviewer #1: Partly

Reviewer #2: Yes

Reviewer #3: Yes

**********

3. Has the statistical analysis been performed appropriately and rigorously? 

Reviewer #1: (No Response)

Reviewer #2: Yes

Reviewer #3: Yes

**********

4. Have the authors made all data underlying the findings in their manuscript fully available?

The PLOS Data policy requires authors to make all data underlying the findings described in their manuscript fully available without restriction, with rare exception (please refer to the Data Availability Statement in the manuscript PDF file). The data should be provided as part of the manuscript or its supporting information, or deposited to a public repository. For example, in addition to summary statistics, the data points behind means, medians and variance measures should be available. If there are restrictions on publicly sharing data—e.g. participant privacy or use of data from a third party—those must be specified.

Reviewer #1: Yes

Reviewer #2: Yes

Reviewer #3: Yes

**********

5. Is the manuscript presented in an intelligible fashion and written in standard English?

PLOS ONE does not copyedit accepted manuscripts, so the language in submitted articles must be clear, correct, and unambiguous. Any typographical or grammatical errors should be corrected at revision, so please note any specific errors here.

Reviewer #1: No

Reviewer #2: Yes

Reviewer #3: Yes

**********

6. Review Comments to the Author

Please use the space provided to explain your answers to the questions above. You may also include additional comments for the author, including concerns about dual publication, research ethics, or publication ethics. (Please upload your review as an attachment if it exceeds 20,000 characters)

Reviewer #1: This research is very interesting and would be a valuable contribution for management of Caribbean reef sharks.

However, I had great difficulty reading through the results section and reconciling figures to text. The numbering is not sequential, there are two figure 1s, and there appears to be more references to figures than actual figures. This could be a result of the formatting by the online system, but none of the printed material matched. The printed figures did not have numbers, so sorting through the paperwork was difficult, and I am still uncertain if I have the figure order correct.

There are a number of other edits that are required. For example, Vemco is now Innovasea and should be referenced correctly. The ethics statement does not match what is stated in the text as having been done (e.g., immature sharks were tagged, not released without tags; duration of restraint is 15-20 minutes in text, but 10 minutes in the ethics statement).

Is there information on tag shedding rates using the method tag implantation in this manuscript. Tag encapsulation and expulsion when placed under the skin is known to occur. This should be addressed in the MS.

Reviewer #2: Abstract

The sentence in line 45/46 should be toned down as less than 60% of the animals were detected after tagging.

Intro

Line 70/71 – I think this sentence could be rephrased to improve readability.

Line 77 – Consider replacing with “Evidence from previous acoustic telemetry studies…”

Line 80 – Home range is an area and therefore it should be stated in km2. However, after reading the full manuscript I realized that the authors are in fact referring to linear home range. Thus “linear” should be added before “home range”.

Line 85 – Consider deleting “…which are…”

M&M

Line 125 – provide the number of receivers.

Fig2 – can be moved to supplements

Range tests (lines 138/142) – The authors should be aware that the range of the VR100 is not equal to the range of the VR2W’s. This should be explicitly mentioned in the text. Also, V9 tags will have a shorter detection range – this should be considered in line with what is assumed in lines 151/156.

Fig3 – can be moved to supplements

Lines 259-261. This is not very clear to me. Did you get duplicate detections (same ID, date and time) on a single receiver? I have never seen or heard about this. Usually duplicates happen in multiple receivers (usually) when there’s some detection range overlap.

Line 285 – “Detections days” – delete the “s” in “detections”.

Lines 292-298 – Please see also (Appert et al., 2023; Kraft et al., 2023)

Lines 299- 311 – See Kraft et al 2023

Line 346 – Authors should be aware that the detection range of the V9 tags (used in sharks <1.1m, representing 1/3 of the detected sharks) was less than 300m. This should be explicitly mentioned in the text. Statistical analysis comparing detection rates and other metrics between animals tagged with V16 and animals tagged with V9 could be interesting to explore. At least to elucidate putative differences in behaviour which might be masked because of the differences in detection range.

Line 355 – With tags transmitting at 90s (on average) it is not possible to obtain hundreds of detections in one hour. Please rephrase this sentence.

Line 464/465 – and what about differences between animals tagged with V16 vs animals tagged with V9?

Fig S1 could be moved to the main document.

Line 560-562 – This could be an artifact (to a certain point) due to the difference in detection range between V16 and V9 tags.

Would it be possible that “passer-by” sharks were captured or died?

Lines 682-690 – See Appert et al 2023 and Kraft et al 2023.

Line 758 – linear home range

Line 826-834 – What are the main environmental differences between summer and winter?

Suggested References

Appert, C., Udyawer, V., Simpfendorfer, C.A., Heupel, M.R., Scott, M., Currey-Randall, L.M., Harborne, A.R., Jaine, F., Chin, A., 2023. Use, misuse, and ambiguity of indices of residence in acoustic telemetry studies. Mar. Ecol. Prog. Ser.

Kraft, S., Gandra, M., Lennox, R.J., Mourier, J., Winkler, A.C., Abecasis, D., 2023. Residency and space use estimation methods based on passive acoustic telemetry data. Movement Ecology 11, 12.

Reviewer #3: Overall this is a well-written manuscript that clearly describes a wide variety of analyses and results. The findings of this paper will be of interest to researchers conducting spatial analysis on highly migratory Caribbean species and include some interesting revelations about Caribbean reef sharks in particular. I only have some minor comments and suggested edits concerning information that could be included in the Abstract and the organization of the figures, which are arranged by section below:

Abstract

- Include a brief description on the types of analyses used to assess the various aspects of space use and site fidelity in the paper.

Tables/Figures

- This is a very figure-heavy manuscript and could benefit from some figures being combined or moved to the supplemental material. My specific recommendations are:

- Figures 9, 10, and 11 could be combined.

- Figure 3 may not be necessary in the main text since these are fairly standard shark sampling gears, so could be moved to supplemental info.

- Figures 1, 2, and 3 are referred to in both the Methods and Results sections, but are different figures in each section.

- Figure numbers only go up to 10 in the text but there are 13 figures associated with the manuscript. Including Figures 1-3 in both the Methods and Results separately would add up to 13 figures, so this may just be a numbering error.

**********

7. PLOS authors have the option to publish the peer review history of their article (what does this mean?). If published, this will include your full peer review and any attached files.

If you choose “no”, your identity will remain anonymous but your review may still be made public.

**Do you want your identity to be public for this peer review?** For information about this choice, including consent withdrawal, please see our Privacy Policy.

Reviewer #1: No

Reviewer #2: No

Reviewer #3: No

**********

[NOTE: If reviewer comments were submitted as an attachment file, they will be attached to this email and accessible via the submission site. Please log into your account, locate the manuscript record, and check for the action link "View Attachments". If this link does not appear, there are no attachment files.]

While revising your submission, please upload your figure files to the Preflight Analysis and Conversion Engine (PACE) digital diagnostic tool, https://pacev2.apexcovantage.com/. PACE helps ensure that figures meet PLOS requirements. To use PACE, you must first register as a user. Registration is free. Then, login and navigate to the UPLOAD tab, where you will find detailed instructions on how to use the tool. If you encounter any issues or have any questions when using PACE, please email PLOS at figures@plos.org. Please note that Supporting Information files do not need this step.

# Author comment: Author response to Decision Letter 1

**DOI:** 10.1371/journal.pone.0293884.r004

17 Oct 2023

PLOS ONE

Dear Editor and Reviewers,

Please find the responses to each point below.

Reviewer #1

The numbering of figures was reviewed and corrected where needed. 

The references to figures in the text were corrected. The figures were cross references in the word document, but it seems this format causes trouble when converted into PDF, unfortunately. Now the numbering was manually entered which hopefully results in correct numbering in the PDF as well. Please note, figures with “S and number” are referring to figures in the Supporting Info.

The references regarding ‘Vemco Ltd’ were amended to ‘Innovasea Systems Inc.’.

The Ethics statement was amended to match the information in the method section. 

We do not have any evidence of tag shedding. Dicken et al.2006 wrote about tag shedding in external tags with an insert point, but we are not aware of any studies suggesting that tags surgically inserted under the skin are shed. Unlike e.g. cat skin, shark skin layers are adhesive allowing the tag to remain in place. It would be pretty difficult for the rounded tag to emerge, the surface of the tag also being of a substance to reduce any reaction by the shark. If the tag had been inserted into the coelum, as some researchers do, then there is a good chance of the tag not only moving, but possibly causing an obstruction. Nevertheless, this is a good point, and it has been addressed in the Method section (starting line 205) and Discussion (starting line 703) to make it clearer. 

Dicken, M. L., Booth, A. J., and Smale, M. J. (2006). Preliminary observations of tag shedding, tag reporting, tag wounds, and tag biofouling for raggedtooth sharks (Carcharias taurus) tagged off the east coast of South Africa. ICES J. Mar. Sci. 63, 1640–1648. doi: 10.1016/j.icesjms.2006.06.009.

Reviewer #2

Line 44/46: The sentence was amended to make it clearer (now line 42-45).

Line 71: The sentence was amended to make it clearer. 

Line 77: The sentence was amended as suggested (now line 79).

Line 80: “linear” was added before “home range”.

Line 85: Sentence was amended as suggested (now line 87).

Line 125: Figure caption was amended to include number of receivers (now line 119).

Fig2: Was moved to supplementary material.

Line 138-142 and 151-156 (range tests): These are good points. The two paragraphs were amended to make this clearer. 

Fig3: Was moved to supplementary material.

Lines 259-261: Relevant section was removed. We did not get any duplicates on the same receiver. It was part of the protocol, but perhaps because the array was not overlapping there was no need for this step. (now line 265)

Line 285: “s” was deleted. (now line 288)

Lines 292-298: Appert et al., 2023 and Kraft et al., 2023 were added to the references. 

Lines 299- 311: The differences that Appert et al., 2023 and Kraft et al 2023 address are briefly mentioned in the Discussion. So instead of discussing the issue of different equations and the consequences for the calculation of RI values here, the relevant section in the Discussion was amended to expand on this topic and to cite both studies (starting line 555). Here, Kraft et al. and Appert et al. were added to the in-text citation (line 304-306).

Line 346: This point is valid and we did consider redoing part of the data analyses, in particular the values for MLD, accounting for a shorter transmission range of the V9 tags. However, after consultation with Innovasea, we decided not to make any changes to the data analyses. While the range would certainly be less for a V9 compared to a V16 tag, the relationship is not linear. That means that *theoretically* an increase of 3dB is double the power but that this does not equate to twice the distance. Hence it is not possible to estimate the V9 range given what we had found for a V16 tag. That being said, we did test for differences between mature and juvenile sharks (most of which were fitted with V9s) and did not find any significant differences–this is mentioned in the MS. We do appreciate the concern raised by the reviewer and to make it clearer, the MS was amended mentioning the difference in transmission range between the V16 and V9 tags in the Methods, Results and Discussion.

Line 355: The sentence was amended to make it clearer.

Line 464/465: The last sentence in the paragraph mentioned that there was no difference between maturity, assuming most of these individuals were fitted with V9 tags. Although this is an interesting and valid point, no changes were made because of the reasons mentioned above.

Fig S1: No changes were made. The overall feedback by reviewers was that MS is ‘figure heavy’. While we believe that the figures are beneficial for the reader to understand and visualize the results, we appreciate the option for readers to view these from the Supporting Information.

Line 560-562: A sentence was added to mention this point. Furthermore, a sentence was added to the Discussion to make it clearer (see line 777).

Would it be possible that “passer-by” sharks were captured or died?: This possibility is mentioned in the Discussion (see line 699). This paragraph was amended to make it clearer. 

Lines 682-690: The suggested references were added. 

Line 758: While this paper uses linear home range, “home range” was not mentioned here. No changes were made.

Line 826-834: A paragraph was added to provide information on Cayman’s summer/winter conditions (starting line 826).

Reviewer #3

Abstract 

Line 44-46: A brief description on the types of analyses used to assess the various aspects of space use and site fidelity was added to the Abstract. 

Tables/Figures: While we believe that the figures are beneficial for the reader to understand and visualize the results, we appreciate the option for readers to view these from the Supporting Information. In response to your specific suggestions, the following changes were made:

-Figures 9, 10, and 11 were kept separate. We believe it would be difficult to see details if all networks would be combined. 

-Figure 3: Was moved to Supporting info. 

-References to Figures 1, 2, and 3 and overall numbering was corrected. The figures were cross references in the word document, but it seems this format caused trouble when converting into PDF, unfortunately. Now the numbering was manually entered which hopefully results in correct numbering in the PDF as well. Please note, figures with “S and number” are referring to figures in the Supporting Info.

PLOS ONE

PLOS Figures: Figures were amended via PACE and the corrected versions were uploaded.

Sincerely,

Dr. Johanna Kohler

Cayman Islands Department of the Environment

Johanna.k.kohler@gmail.com, +1 (345) 926-0135

Attachment Submitted filename: PLOS ONE edits_response to reviewers_final.docx Click here for additional data file.

# Peer review: Decision Letter 2

**DOI:** 10.1371/journal.pone.0293884.r005

**Authors:** Rui Coelho

23 Oct 2023

Individual residency behaviours and seasonal long-distance movements in acoustically tagged Caribbean reef sharks in the Cayman Islands

PONE-D-23-13313R2

Dear Dr. Kohler,

We’re pleased to inform you that your manuscript has been judged scientifically suitable for publication and will be formally accepted for publication once it meets all outstanding technical requirements.

Within one week, you’ll receive an e-mail detailing the required amendments. When these have been addressed, you’ll receive a formal acceptance letter and your manuscript will be scheduled for publication.

An invoice for payment will follow shortly after the formal acceptance. To ensure an efficient process, please log into Editorial Manager at http://www.editorialmanager.com/pone/, click the 'Update My Information' link at the top of the page, and double check that your user information is up-to-date. If you have any billing related questions, please contact our Author Billing department directly at authorbilling@plos.org.

If your institution or institutions have a press office, please notify them about your upcoming paper to help maximize its impact. If they’ll be preparing press materials, please inform our press team as soon as possible -- no later than 48 hours after receiving the formal acceptance. Your manuscript will remain under strict press embargo until 2 pm Eastern Time on the date of publication. For more information, please contact onepress@plos.org.

Kind regards,

Rui Coelho, PhD

Academic Editor

PLOS ONE

Additional Editor Comments (optional):

Dear Dr. Kohler,

Thank you for the submission of the revised paper “Individual residency behaviours and seasonal long-distance movements in acoustically tagged Caribbean reef sharks in the Cayman Islands” for consideration to publication in PlosOne.

The concerns that were raised by the referees in the previous version of the paper have been addressed. I suggest acceptance of the paper.

Best regards,

Rui Coelho

Reviewers' comments:

Reviewer's Responses to Questions

**Comments to the Author**

1. If the authors have adequately addressed your comments raised in a previous round of review and you feel that this manuscript is now acceptable for publication, you may indicate that here to bypass the “Comments to the Author” section, enter your conflict of interest statement in the “Confidential to Editor” section, and submit your "Accept" recommendation.

Reviewer #1: All comments have been addressed

**********

2. Is the manuscript technically sound, and do the data support the conclusions?

The manuscript must describe a technically sound piece of scientific research with data that supports the conclusions. Experiments must have been conducted rigorously, with appropriate controls, replication, and sample sizes. The conclusions must be drawn appropriately based on the data presented. 

Reviewer #1: Yes

**********

3. Has the statistical analysis been performed appropriately and rigorously? 

Reviewer #1: I Don't Know

**********

4. Have the authors made all data underlying the findings in their manuscript fully available?

The PLOS Data policy requires authors to make all data underlying the findings described in their manuscript fully available without restriction, with rare exception (please refer to the Data Availability Statement in the manuscript PDF file). The data should be provided as part of the manuscript or its supporting information, or deposited to a public repository. For example, in addition to summary statistics, the data points behind means, medians and variance measures should be available. If there are restrictions on publicly sharing data—e.g. participant privacy or use of data from a third party—those must be specified.

Reviewer #1: Yes

**********

5. Is the manuscript presented in an intelligible fashion and written in standard English?

PLOS ONE does not copyedit accepted manuscripts, so the language in submitted articles must be clear, correct, and unambiguous. Any typographical or grammatical errors should be corrected at revision, so please note any specific errors here.

Reviewer #1: Yes

**********

6. Review Comments to the Author

Please use the space provided to explain your answers to the questions above. You may also include additional comments for the author, including concerns about dual publication, research ethics, or publication ethics. (Please upload your review as an attachment if it exceeds 20,000 characters)

Reviewer #1: (No Response)

**********

7. PLOS authors have the option to publish the peer review history of their article (what does this mean?). If published, this will include your full peer review and any attached files.

If you choose “no”, your identity will remain anonymous but your review may still be made public.

**Do you want your identity to be public for this peer review?** For information about this choice, including consent withdrawal, please see our Privacy Policy.

Reviewer #1: No

**********

# Editor report: Acceptance letter

**DOI:** 10.1371/journal.pone.0293884.r006

**Authors:** Rui Coelho

15 Nov 2023

PONE-D-23-13313R2 

Individual residency behaviours and seasonal long-distance movements in acoustically tagged Caribbean reef sharks in the Cayman Islands 

Dear Dr. Kohler:

I'm pleased to inform you that your manuscript has been deemed suitable for publication in PLOS ONE. Congratulations! Your manuscript is now with our production department. 

If your institution or institutions have a press office, please let them know about your upcoming paper now to help maximize its impact. If they'll be preparing press materials, please inform our press team within the next 48 hours. Your manuscript will remain under strict press embargo until 2 pm Eastern Time on the date of publication. For more information please contact onepress@plos.org.

If we can help with anything else, please email us at customercare@plos.org.

Thank you for submitting your work to PLOS ONE and supporting open access. 

Kind regards, 

PLOS ONE Editorial Office Staff

on behalf of

Dr. Rui Coelho 

Academic Editor

PLOS ONE
